# LncRNA CDKN2B-AS1 stabilized by IGF2BP3 drives the malignancy of renal clear cell carcinoma through epigenetically activating NUF2 transcription

**DOI:** 10.1038/s41419-021-03489-y

**Published:** 2021-02-19

**Authors:** Xina Xie, Jiatian Lin, Xiaoqin Fan, Yuantang Zhong, Yequn Chen, Kaiqing Liu, Yonggang Ren, Xiangling Chen, Daihuan Lai, Xuyi Li, Zesong Li, Aifa Tang

**Affiliations:** 1 Guangdong Key Laboratory of Systems Biology and Synthetic Biology for Urogenital Tumors, Shenzhen Second People’s Hospital, First Affiliated Hospital of Shenzhen University, 518000 Shenzhen, Guangdong China; 2 Department of Minimally Invasive Intervention, Peking University Shenzhen Hospital, 518000 Shenzhen, Guangdong China; 3 Department of Otolaryngology, Shenzhen Second People’s Hospital, First Affiliated Hospital of Shenzhen University, 518000 Shenzhen, Guangdong China; 4 Department of Urology, Longgang District Central Hospital, 518100 Shenzhen, Guangdong China; 5 Department of Community Surveillance, The First Affiliated Hospital of Shantou University Medical College, 515041 Shantou, Guangdong China

**Keywords:** Long non-coding RNAs, Renal cell carcinoma

## Abstract

Because of the lack of sensitivity to radiotherapy and chemotherapy, therapeutic options for renal clear cell carcinoma (KIRC) are scarce. Long noncoding RNAs (lncRNAs) play crucial roles in the progression of cancer. However, their functional roles and upstream mechanisms in KIRC remain largely unknown. Exploring the functions of potential essential lncRNAs may lead to the discovery of novel targets for the diagnosis and treatment of KIRC. Here, according to the integrated analysis of RNA sequencing and survival data in TCGA-KIRC datasets, cyclin-dependent kinase inhibitor 2B antisense lncRNA (CDKN2B-AS1) was discovered to be the most upregulated among the 14 lncRNAs that were significantly overexpressed in KIRC and related to shorter survival. Functionally, CDKN2B-AS1 depletion suppressed cell proliferation, migration, and invasion both in vitro and in vivo. Mechanistically, CDKN2B-AS1 exerted its oncogenic activity by recruiting the CREB-binding protein and SET and MYND domain-containing 3 epigenetic-modifying complex to the promoter region of Ndc80 kinetochore complex component (NUF2), where it epigenetically activated NUF2 transcription by augmenting local H3K27ac and H3K4me3 modifications. Moreover, we also showed that CDKN2B-AS1 interacted with and was stabilized by insulin-like growth factor 2 mRNA-binding protein 3 (IGF2BP3), an oncofetal protein showing increased levels in KIRC. The Kaplan–Meier method and receiver operating curve analysis revealed that patients whose IGF2BP3, CDKN2B-AS1 and NUF2 are all elevated showed the shortest survival time, and the combined panel (containing IGF2BP3, CDKN2B-AS1, and NUF2) possessed the highest accuracy in discriminating high-risk from low-risk KIRC patients. Thus, we conclude that the stabilization of CDKN2B-AS1 by IGF2BP3 drives the malignancy of KIRC through epigenetically activating NUF2 transcription and that the IGF2BP3/CDKN2B-AS1/NUF2 axis may be an ideal prognostic and diagnostic biomarker and therapeutic target for KIRC.

## Introduction

Renal cell carcinoma (RCC) is the second most common urological malignancy, accounting for 90% of kidney tumors, of which ~80–90% are renal clear cell carcinomas (KIRC)^1^. In 2020, there have been nearly 73,750 new RCC cases and 14,830 deaths caused by RCC in the USA^2^. Although localized or locally advanced RCC can be cured by surgical resection, ~30% of the patients still show local recurrence or distant metastasis within 5 years, and the 5-year survival rate is only 10% (refs. ^3–5^). Owing to the lack of sensitivity to conventional radiotherapy and chemotherapy, immunotherapy and targeted therapy are used as the first-line treatments for KIRC patients with metastasis, but the outcomes are very poor^4,6,7^. This is mainly due to the fact that the etiology and pathogenesis of KIRC have not yet been fully clarified, and clinically effective therapeutic targets for KIRC are very limited. Therefore, it is of utmost importance to improve our understanding of the molecular mechanisms underlying KIRC pathogenesis, and identify novel sensitive and reliable parameters that potentially serve as diagnostic biomarkers and therapeutic targets for the treatment of patients with KIRC^8^.

In 2018, the first RNAi drug (Patisiran)^9^ was approved by the U.S. FDA for the treatment of hereditary transthyretin-mediated amyloidosis, further confirming the reliability of nucleic acids as targets for the disease treatment. In fact, nucleic acid drugs, including ribonucleic acid and oligonucleotide drugs, can not only work in cells as conventional small-molecule drugs but can also precisely target the nucleus, achieving effects that traditional drugs cannot. Thus, nucleic acid drugs may help resolve the current lack of targeted drugs for KIRC. Long noncoding RNAs (lncRNAs), a class of RNA molecules that are >200 nucleotides in length and have little or no protein-coding function, have been confirmed to be closely related to the tumorigenesis and progression of various cancers^10,11^. Over the past decade, extensive molecular research has shown that some lncRNAs can function as oncogenes and serve as biomarkers for the clinical diagnosis and prognosis of cancer, such as H19, HOTAIR, PVT1, CCAT, UCA1, MALAT1, and XIST^12^. Consequently, lncRNAs are expected to become a promising target of nucleic acid drugs in cancer therapy, and it is important to study the functions of potential essential lncRNAs in controlling cancer initiation and progression in depth.

Therefore, this study aimed to uncover more potential essential lncRNAs that might affect the prognosis of KIRC and identify new diagnostic biomarkers and therapeutic targets for KIRC treatment. In a systematic screen, among the 14 lncRNAs that were significantly overexpressed and related to shorter survival in KIRC, cyclin-dependent kinase inhibitor 2B antisense lncRNA (CDKN2B-AS1) was discovered to be the most upregulated. We then verified the functional roles and molecular process of CDKN2B-AS1 in KIRC malignancy and highlighted its clinical implication. We also demonstrated the upstream mechanism underlying its upregulation in KIRC.

## Materials and methods

### Data mining and analysis

The differentially expressed lncRNAs between KIRC tissues and normal tissues from RNA-seq in TCGA dataset were analyzed using the circlncRNAnet database (http://120.126.1.61/circlnc/circlncRNAnet/lncRNA_TCGA/index.php, access date: 2018.02.08, false discovery rate = 0.05, and a fold-change cutoff = 2)^13^. The survival data in TCGA-KIRC dataset were acquired from the OncoLnc website (http://www.oncolnc.org/, access date: 2018.02.08). The RNA expression of CDKN2B-AS1, Ndc80 kinetochore complex component (NUF2), and insulin-like growth factor 2 mRNA-binding protein 3 (IGF2BP3) was profiled based on normalized RNA-seq data in TCGA-KIRC dataset (TCGA_KIRC_exp_HiSeqV2–2015–02–24) from the UCSC Xena Browser (https://xenabrowser.net/datapages/). The mRNAs co-expressed with CDKN2B-AS1 in TCGA-KIRC cases were identified using the TANRIC database (https://ibl.mdanderson.org/tanric/_design/basic/query.html, access date: 2019.03.10)^14^ and circlncRNAnet (http://120.126.1.61/circlnc/circlncRNAnet/lncRNA_TCGA/index.php, access date: 2018.04.19)^13^. The potential target genes of CDKN2B-AS1 were predicted using the LncMAP database (http://bio-bigdata.hrbmu.edu.cn/LncMAP/, access date: 2018.03.07)^15^. The binding potential of IGF2BPs to CDKN2B-AS1 was analyzed using the RNA–Protein Interaction Prediction (RPISeq) database (http://pridb.gdcb.iastate.edu/RPISeq/).

### Collection of KIRC tissues

A total of 42 pairs of KIRC tissues and matched adjacent normal tissues were obtained from patients admitted to the First Affiliated Hospital of Shantou University Medical College. All subjects provided written informed consent. The study was conducted in accordance with the Declaration of Helsinki, and the protocol was approved by the Ethics Committee of the First Affiliated Hospital of Shantou University Medical College (number: 2020-031). All the tissue samples were obtained directly from surgery after removal of necessary amount of tissue for routine pathology examination and confirmed for KIRC.

### Cell culture and treatment

Human KIRC cell lines 769-P, ACHN, 786-O, Caki-1, and Caki-2 were obtained from the American Type Culture Collection (Rockville, MD, USA). Caki-1 and Caki-2 cells were cultured in McCoy’s 5A medium (Gibco, USA), 769-P and 786-O cells were cultured in RPMI-1640 medium (Hyclone, USA), and ACHN cells were cultured in Eagle’s MEM medium (Gibco, USA). All media were supplemented with 10% fetal bovine serum (FBS; Gibco, USA) and all cell lines were cultured in a humidified incubator at 37 °C with 5% CO_2_. The cell lines were authenticated and characterized by the supplier and were monitored regularly for their authenticity (Biowing Applied Biotechnology Co. Ltd, Shanghai, China) and to be free of mycoplasma contamination.

Small interfering RNAs (siRNAs) and antisense oligonucleotides (ASOs) for CDKN2B-AS1, CREB-binding protein (CBP), SET and MYND domain-containing 3 (SMYD3), and IGF2BP3 were obtained from RiboBio Co. (Guangzhou, China), and were transfected using Lipofectamine RNAiMAX (Invitrogen, USA) according to the manufacturer’s instructions. The pcDNA3.1-NUF2 plasmid was obtained from Transheep (Shanghai, China) and transfected at a final concentration of 3.0 μg/mL using FuGENE^®^ HD Reagent (Promega, Madison, WI, USA), according to the manufacturer’s instructions. For actinomycin D RNA stability assays, the cells were treated at the indicated time points with 5 μg/mL actinomycin D (Sigma, St Louis, MO, USA). siRNA and ASO primer sequences are specified in Supplementary Table S1.

### qRT-PCR

Total RNA was extracted from tissues and cells using Trizol (Invitrogen, USA), and was reverse-transcribed using a PrimeScript RT reagent Kit with gDNA Eraser (Takara Bio, Inc., Japan) for cDNA synthesis and genomic DNA removal. qRT-PCR was performed using a QuantiNova^TM^ SYBR Green PCR mix kit (QIAGEN, Germany) and carried out in the Applied Biosystems Prism 7500. The relative expression levels of the analyzed genes were compared to those of β-actin, and fold changes were calculated using the 2^−∆∆Ct^ method for cell experiments and the 2^−∆Ct^ method for tissue samples^16^. All qRT-PCR primers used are listed in Supplementary Table S1.

### Western blot analysis

Human KIRC cell lines were lysed in RIPA buffer supplemented with 1% protease inhibitor cocktail (Millipore, USA). Protein concentrations were determined using a BCA protein assay kit (Beyotime, China). Then, 30 μg of the denatured protein samples were subjected to 8% SDS–PAGE and transferred to a PVDF membrane (Millipore, USA). Immunoblotting involved primary antibodies against NUF2 (1:800; No. 15731-1-AP; Proteintech, USA), CBP (1:1000; No. PA1-847; Thermo Fisher, USA), SMYD3 (1:1000; No. ABE2870; Millipore, USA), IGF2BP3 (1:1000; No. 14642-1-AP; Proteintech, USA), and glyceraldehyde 3-phosphate dehydrogenase (GAPDH; 1:10,000; No. 60004-1-Ig; Proteintech, USA). Corresponding secondary antibodies were applied, and blots were developed using Super ECL Plus Detection Reagent (NovasyGen, China). The intensity of bands was determined with ImageJ 2X software (National Institutes of Health, Bethesda, MD, USA) and normalized to that of GAPDH.

### Cell nuclear and cytoplasmic RNA isolation

The cytoplasmic and nuclear RNAs of 769-P and ACHN cells were separated using the PARIS kit (Life Technologies, USA), according to the manufacturer’s instructions. The expression levels of CDKN2B-AS1, U3, and GAPDH in the nucleus or cytoplasm were detected by qRT-PCR.

### Chromatin immunoprecipitation assay

As previously described^17^, chromatin immunoprecipitation (ChIP) was performed using EZ-Magna ChIP A and EZ-Magna ChIP G Kits (Millipore, USA), following the manufacturer’s instructions. Briefly, ChIP grade antibodies were as follows: anti-histone H3 lysine 27 acetylation (H3K27ac; Thermo Fisher, USA), anti-histone H3 lysine 4 trimethylation (H3K4me3; Millipore, USA), anti-histone *H3* lysine 27 trimethylation (H3K27me3; Millipore, USA), anti-P300 (Santa Cruz Biotechnology, USA), anti-CBP (Thermo Fisher, USA), anti-SMYD3 (Millipore, USA), and normal IgG (Millipore, USA). Immunoprecipitated DNA was amplified by qPCR, and normalized to the input DNA. Primer sequences for the promoter region of *NUF2* are listed in Supplementary Table S1.

### ChIP-seq data analyses

ChIP-seq data of KIRC samples and normal tissues were obtained from the NCBI Gene Expression Omnibus (GEO) datasets (https://www.ncbi.nlm.nih.gov/gds/). The BED files were downloaded from GEO datasets and were visualized using the WashU EpiGenome Browser (https://epgg-test.wustl.edu/browser/). The information on ChIP-seq data is listed in Supplementary Table S2.

### RNA immunoprecipitation assay

As previously described^17^, the Magna RIP RNA-Binding Protein Immunoprecipitation Kit (Millipore, USA) was used to determine the relationship between CDKN2B-AS1 and CBP, SMYD3, or IGF2BP3 according to the manufacturer’s instructions. Antibodies used for the RNA immunoprecipitation (RIP) assay were as follows: anti-CBP, anti-SMYD3, anti-IGF2BP3, and anti-lgG antibodies (5 μg per reaction). Coprecipitated RNAs were used for cDNA synthesis and were evaluated by qRT-PCR.

### Cell proliferation, colony formation, and 5-ethynyl-2′-deoxyuridine incorporation assays

The ability of cell was detected using the Cell Counting Kit-8 (CCK-8; Dojindo, Kumamoto, Japan) according to the manufacturer’s instructions. A total of 5 × 10^3^cells/well were plated into 96-well plates after transfection for 24 h, and 10 μL of CCK-8 solution (Dojindo, Japan) was added on days 1, 2, and 3, respectively. Subsequently, the cells were incubated for 2 h at 37 °C, and the optical density values were measured at 450 nm using a microplate reader (Bio‑Rad Laboratories, Inc.). For the colony formation assay, transfected cells were seeded in six-well plates at 2 × 10^3^ per well. After 10–14 days, cell colonies were fixed with 4% paraformaldehyde, air dried, and stained with 0.05% crystal violet (Beyotime, Shanghai, China). The colonies were imaged and counted. For the 5-ethynyl-2′-deoxyuridine (EdU) incorporation assay, transfected cells were seeded into 96-well plates. The EdU incorporation assay kit (RiboBio, China) was used to evaluate cell proliferation according to the manufacturer’s instructions. Images were acquired under a fluorescent microscope at 567 nm excitation.

### Wound healing and transwell assays

Transfected cells were seeded in six-well plates and cultured until 100% confluence. The cell monolayer was scraped in a straight line using a 10 μL pipette tip to create an artificial scratch, washed with PBS twice, and the medium was replaced with serum-free medium. Images were captured at 0, 6, 12, and 24 h for 769-P cells and 0, 24, 48, and 72 h for ACHN cells. Cell healing rates were calculated based on the fraction of cell coverage across the line. Cell migration and invasion abilities were measured using transwell chambers (8-μm pore size, BD Biosciences, USA). In the migration assay, 5 × 10^4^ transfected cells cultured in 0.5% FBS medium were seeded onto the upper chambers of the transwell, and medium with 20% FBS was added to the lower chamber. After 24 h of incubation, the chamber was fixed with 4% paraformaldehyde and stained using 0.05% crystal violet (Beyotime, Shanghai, China). Cells migrated through the pores were imaged and counted. For the invasion assay, the membrane was precoated with Matrigel and 1.0 × 10^5^ transfected cells were added to the top chamber.

### Immunohistochemistry analysis and scoring methods

Paraffin-embedded KIRC tissue microarrays (No. HkidE180Su02) were purchased from Shanghai Biochip Company Ltd. (Shanghai, China). The sections were dewaxed in xylene and rehydrated in different concentrations of ethanol. Endogenous peroxidase was blocked by 3% H_2_O_2_ and microwave heating was performed for antigen retrieval. After blocking nonspecific antigen binding with 3% BSA at 25 °C for 1 h, the sections were incubated with a specific primary antibody against IGF2BP3 (1:200; No. 14642-1-AP; Proteintech, USA) at 4 °C overnight. After incubation with the HRP-conjugated secondary antibody at 37 °C for 1 h, the sections were counterstained with hematoxylin and stained with diaminobenzidine. Images were taken using a Nikon ECLIPSE Ti microscope (Fukasawa, Japan). The immunostaining intensity of each sample was graded as negative = 0, weak = 1, moderate = 2, or strong = 3. The proportion of positively stained cells was graded as negative = 0, 0–25% = 1, 26–50% = 2, 51–75% = 3, or 76–100% = 4. The immunostaining score was calculated as the sum of the intensity score and positive rate score.

### CDKN2B-AS1 short hairpin RNA and NUF2 overexpression lentiviral vector transduction

The lentiviral vectors used to overexpress NUF2 and knockdown CDKN2B-AS1 were constructed by OBIO Co. (Shanghai, China) and GenePharma Co. (Shanghai, China), respectively. Human full-length cDNA of NUF2 was cloned into the expression vector pLenti-CMV-EGFP-3FLAG-PGK-Zeo, named pLenti-NUF2, and the empty lentiviral expression vector was used as control (pLenti-vector). The short hairpin RNA sequence (5′-GGTCATCTCATTGCTCTAT-3′)^18^ targeting human CDKN2B-AS1 was inserted into the lentiviral pGLV3-CMV-EGFP-T2A-Puro vector, designated pGLV3-shCDKN2BAS1, and the negative control (NC) was named pGLV3-shNC. ACHN cells were infected for 72 h with pGLV3-shCDKN2BAS1 or pGLV3-shNC, respectively. Puromycin (Sangon Biotech, China) was used to select stable cell colonies and the stable cell lines were reinfected for 72 h with pLenti-NUF2 or pLenti-vector, respectively. Stable cell colonies were screened using zeocin (Sangon Biotech, China).

### Animal models

Male BALB/c nude mice (4–6 weeks old) were purchased from Vital River Laboratory Animal Technology Co. (Beijing, China). To detect the effects of CDKN2B-AS1 and NUF2 on the tumorigenicity of ACHN cells, ACHN cells (5 × 10^6^) pre-infected with different lentiviral vectors were randomized subcutaneously injected into the flank of BALB/c nude mouse (*n* = 6 or 5 per group). Tumor sizes were measured every 4 days using a Vernier caliper, and the tumor volume was calculated as length × width/2. Mice were sacrificed after injection for 44 days and their tumor tissues were further examined by qRT-PCR. Tumor measurements and statistical analysis were performed by researchers who were blinded for the control and treatment groups. All animal experiments were approved by the Animal Experimental Ethics Committee of Shenzhen University.

### Statistical analysis

Statistical analysis was performed using SPSS 23.0 (SPSS Inc., Chicago, IL, USA). The differences between groups for cell and animal experiments were analyzed using an independent sample *t* test, one-way ANOVA, Student–Newman–Keuls test, or Dunnett multiple comparison test as appropriate. The expression of CDKN2B-AS1 in KIRC tissues and matched adjacent normal tissues was analyzed by a paired-sample *t* test. Survival curves were estimated using the Kaplan–Meier method and compared using log-rank tests. Survival data were evaluated using univariate and multivariate Cox regression analyses. Pearson correlation analysis was used to evaluate the association between different factors. Receiver operating curve (ROC) analysis was performed to evaluate the potential of CDKN2B-AS1, NUF2, and IGF2BP3 to differentiate high-risk from low-risk KIRC patients showing area under the curve and 95% confidence interval (CI). All data are shown as the mean ± SEM. Statistical significance was defined as *p* < 0.05.

## Results

### Elevated expression of CDKN2B-AS1 was correlated with poor outcome in patients with KIRC

**Fig. 1:**
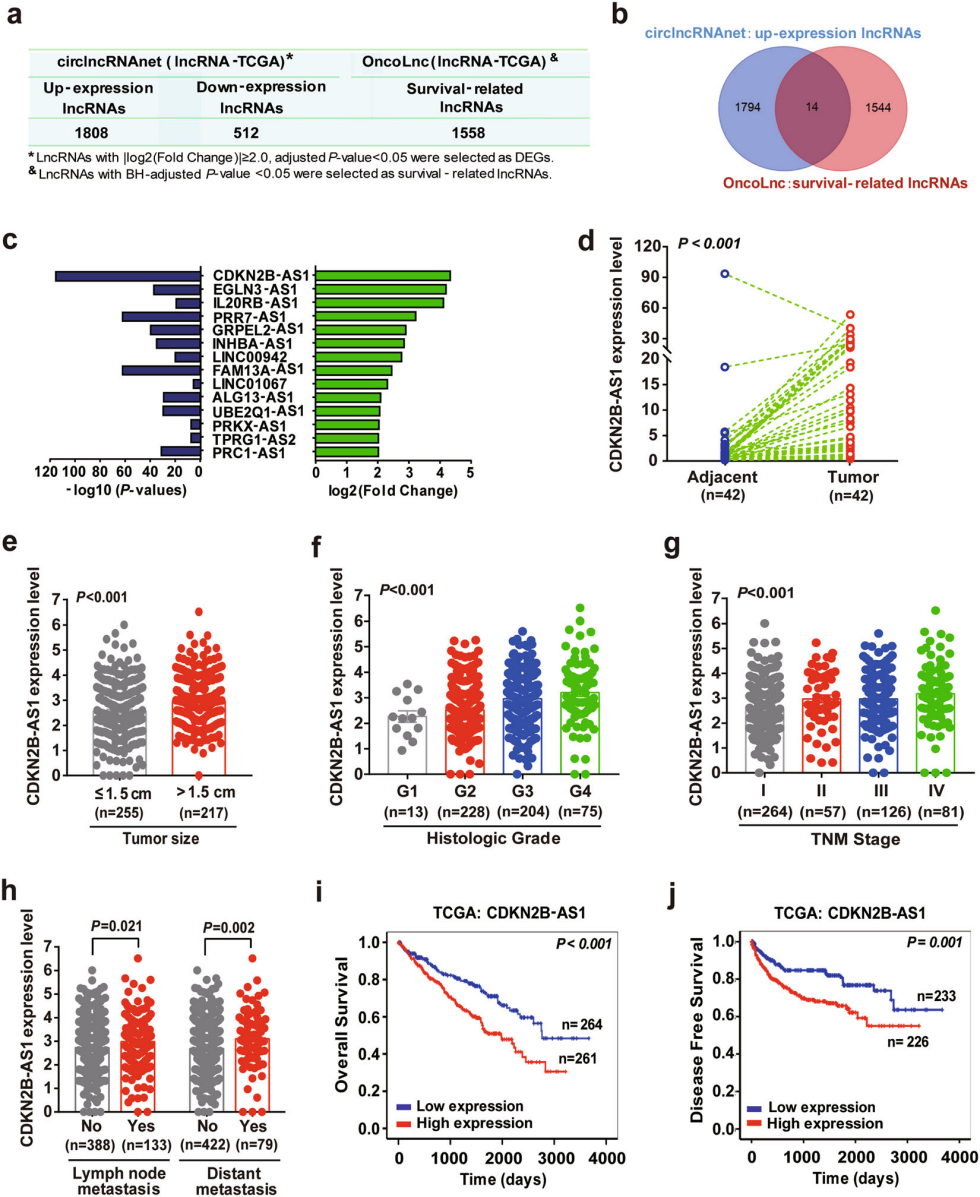
Identification of an essential lncRNA CDKN2B-AS1 whose overexpression is associated with poor prognosis of KIRC. **a** Analysis of lncRNAs which were differentially expressed and related to KIRC survival in TCGA, using circlncRNAnet and OncoLnc database, respectively. **b** Venn diagram presenting the lncRNAs that were upregulated (1808 lncRNAs), related to survival (1558 lncRNAs), or both (14 lncRNAs) in KIRC. **c** The 14 lncRNAs which were upregulated and related to a shorter survival in KIRC. **d** qRT-PCR analysis of CDKN2B-AS1 expression in 42 paired KIRC tissues (tumor) and adjacent normal tissues (adjacent); β-actin was used as the internal control. **e**–**h** CDKN2B-AS1 expression was analyzed in KIRC patients regarding tumor size (**e**), histologic grade (**f**), TNM stage (**g**), and metastasis status (**h**), using RNA-seq data in TCGA. Error bars represent the SEM. **i**, **j** Kaplan–Meier analysis showed the overall survival and disease-free survival in KIRC patients based on the expression of CDKN2B-AS1 in TCGA dataset, the high and low expression groups were divided by the median of CDKN2B-AS1 levels. *P* value calculated by paired-sample *t* test (**d**), independent sample *t* test (**e**, **h**), one-way ANOVA (**f**, **g**), or log-rank test (**i**, **j**).

To identify the important lncRNAs that potentially drove KIRC progression, we detected a total of 1808 upregulated lncRNAs and 512 downregulated lncRNAs in KIRC tissues compared to normal tissues in TCGA datasets, using circlncRNAnet database (Fig. 1a). Meanwhile, we obtained 1558 lncRNAs that were related to survival in KIRC patients from OncoLnc database, and 14 lncRNAs that were both significantly overexpressed and related to a shorter survival (Fig. 1a, b). Of these, CDKN2B-AS1, with a 4.32-log2 [fold change], was the most upregulated lncRNA in KIRC tissues (Fig. 1c). Next, qRT-PCR analysis of CDKN2B-AS1 in 42 pairs of KIRC and matched tumor-adjacent tissues revealed that CDKN2B-AS1 was prominently higher in tumor tissues (Fig. 1d).

Furthermore, TCGA dataset revealed that CDKN2B-AS1 was highly expressed in the tissues of patients with a large tumor size, higher tumor grades, advanced tumor node metastasis (TNM) stages, and metastasis (Fig. 1e–h and Supplementary Table S3). Moreover, the Kaplan–Meier survival curve showed that KIRC patients with high CDKN2B-AS1 expression had markedly shorter overall survival (OS), and disease-free survival (DFS) rates than those with low CDKN2B-AS1 expression (Fig. 1i, j). In addition, age, histological grade, TNM stage, tumor invasion, distant metastasis, tumor size, and CDKN2B-AS1 levels appeared to be correlated with the survival period of KIRC patients (Supplementary Table S4). The multivariate analysis further demonstrated that CDKN2B-AS1 could serve as an independent prognostic factor for worse OS among KIRC patients (HR = 1.174, 95% CI: 1.002–1.375, *p* = 0.047; Supplementary Table S4). Therefore, our data revealed that high CDKN2B-AS1 expression correlated with poor clinical outcomes in KIRC patients.

### Depletion of CDKN2B-AS1 suppresses KIRC growth and metastasis both in vitro and in vivo

**Fig. 2:**
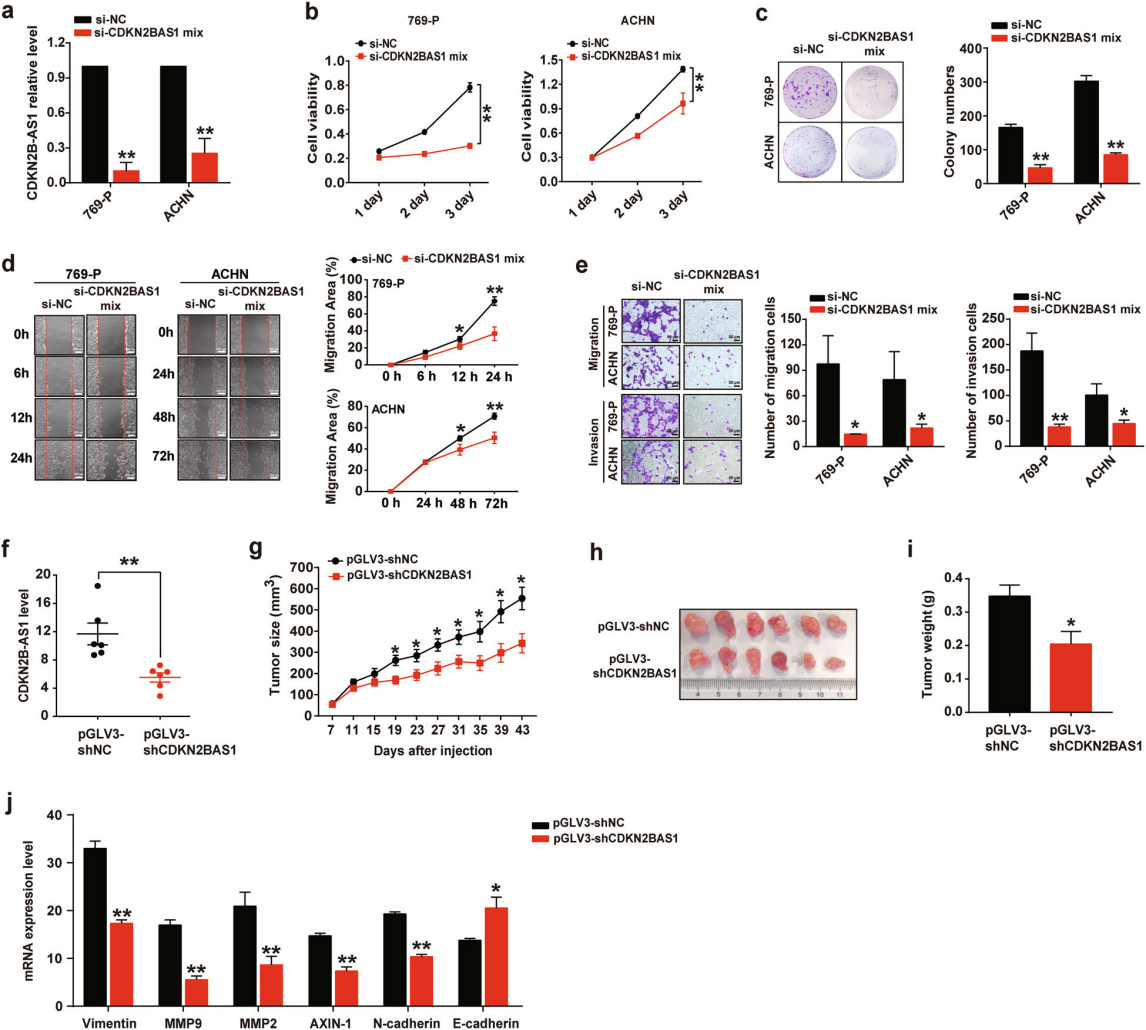
Depletion of CDKN2B-AS1 suppresses KIRC growth and metastasis in vitro and in vivo. **a** qRT-PCR analysis of CDKN2B-AS1 expression in 769-P and ACHN cells transfected with siRNAs and ASOs against CDKN2B-AS1 (si-CDKN2B-AS1 mix) for 48 h, β-actin was used as the internal control. **b**, **c** Cell Counting Kit‑8 (CCK-8) and colony formation assays were used to determine cell viability and proliferation in 769-P and ACHN cells transfected with the si-CDKN2B-AS1 mix or si‑negative control (NC). **d**, **e** Wound healing and transwell assays were performed to assess the cell migration and invasion, ImageJ software was used for migration area calculation and cell counting. Scale bar: 200 μm (**d**) or 50 μm (**e**). **f**–**j** ACHN cells pre-infected with lentiviral were subcutaneously injected into the flank of each BALB/c nude mouse (*n* = 6 per group). qRT-PCR analysis of CDKN2B-AS1, vimentin, MMP9, MMP2, AXIN-1, N-cadherin, and E-cadherin expression in tumor tissues, β-actin was used as the internal control (**f**, **j**); growth curves of tumors in the nude mice (**g**); tumor photographs (**h**); and tumor weight (**i**). Error bars represent the SEM. **p < 0.01 vs. si-NC or pGLV3-shNC, *p < 0.05 vs. si-NC or pGLV3-shNC. Data are representative from three independent experiments, *p* value calculated by independent sample *t* test.

Since CDKN2B-AS1 expression was associated with tumor size and metastasis, we next validated the effects of CDKN2B-AS1 in KIRC cell proliferation and metastasis. Firstly, we found that KIRC cell lines 769-P and ACHN showed higher levels of CDKN2B-AS1 than Caki-1, Caki-2, and 786-O cells (Supplementary Fig. S1). Then, we knocked down CDKN2B-AS1 (si-CDKN2B-AS1 mix, including ASO1, ASO3, siRNA1, and siRNA3 against CDKN2B-AS1; Supplementary Fig. S2) in 769-P and ACHN cells, and demonstrated that CDKN2B-AS1 depletion led to the significant suppression of cell viability over a 3-day culture (Fig. 2a, b). Consistently, colony formation assays indicated that CDKN2B-AS1 knockdown significantly reduced the number of colonies formed by ~75% and 71% in 769-P and ACHN cells, respectively, compared with si‑NC‑transfected cells (Fig. 2c). In addition, wound healing and transwell assays demonstrated that CDKN2B-AS1 suppression also considerably attenuated the migratory and invasive capacities of KIRC cells (Fig. 2d, e).

Moreover, CDKN2B-AS1 expression was decreased in the tumor tissues of mice injected with CDKN2B-AS1-depleted ACHN cells (Fig. 2f). As expected, mice in the CDKN2B-AS1-knockdown group exhibited significantly smaller tumor sizes and tumor weights than those in the control group (Fig. 2g–i). Furthermore, we also observed that mice injected with CDKN2B-AS1-depleted cells had lower expression of vimentin, matrix metalloprotein 9 (MMP9), matrix metalloprotein 2 (MMP2), AXIN-1, N-cadherin, and higher expression of E-cadherin levels than mice injected with control cells (Fig. 2j). Therefore, our results indicated that CDKN2B-AS1 inhibition may be an effective way to diminish KIRC growth and metastasis, thereby decreasing KIRC malignancy in vitro and in vivo.

### CDKN2B-AS1 contributes to KIRC progression via NUF2

**Fig. 3:**
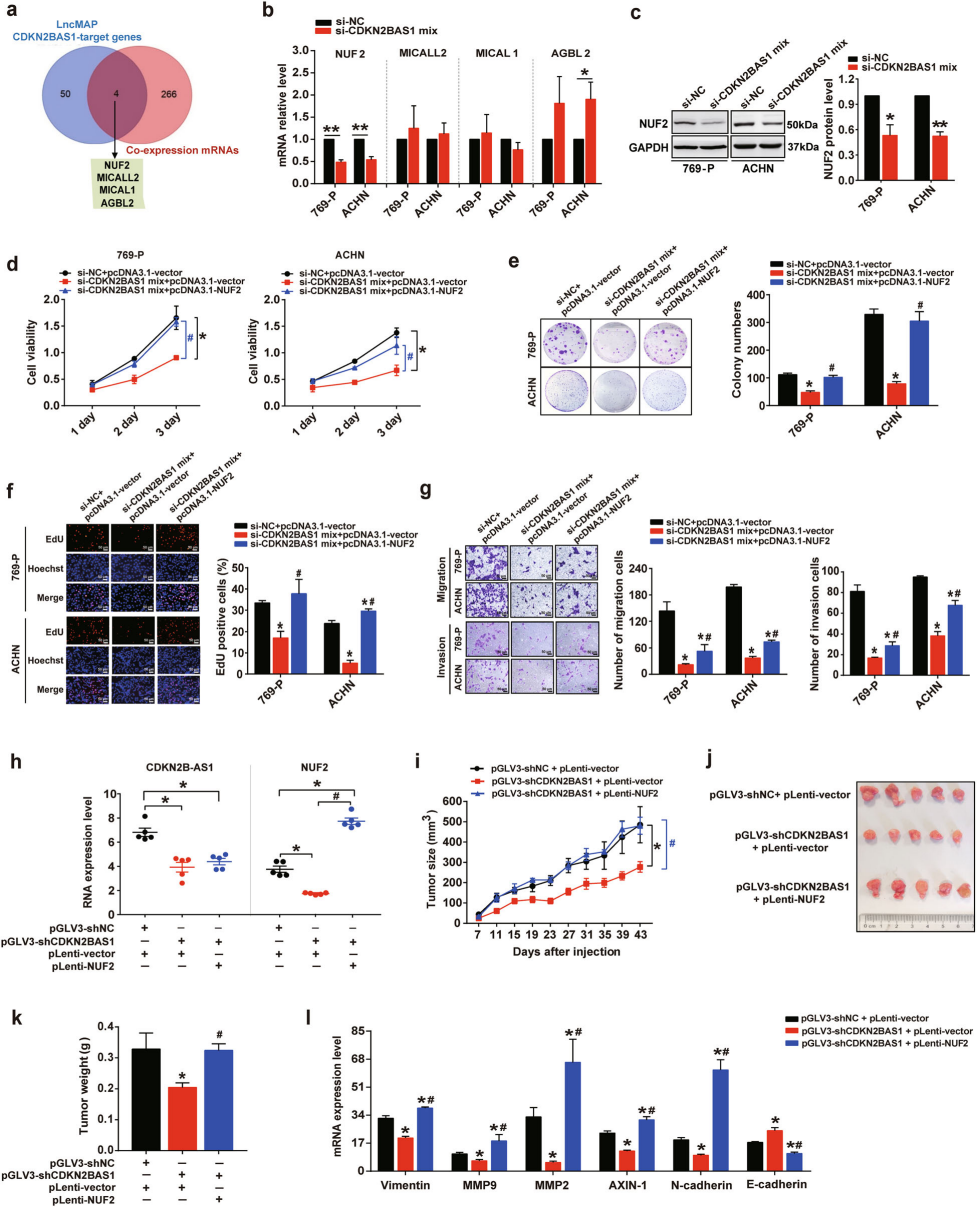
CDKN2B-AS1 contributes to KIRC progression via NUF2 in vitro and in vivo. **a** Venn diagram showing the genes that may be regulated by CDKN2B-AS1 (54 genes), co-expressed with CDKN2B-AS1 (270 genes), or both (4 genes). **b**, **c** qRT-PCR analysis of NUF2, MICALL2, MICAL1, and AGBL2 expression in 769-P and ACHN cells transfected with the si-CDKN2B-AS1 mix for 48 h, β-actin was used as the internal control (**b**); western blot analysis of NUF2 protein expression with GAPDH as the internal control and quantitation of protein levels performed using densitometry (**c**). **d**–**g** 769-P and ACHN cells were transfected with si-CDKN2B-AS1 mix plus pcDNA3.1-NUF2 plasmid for 48 h; CCK-8 (**d**), colony formation (**e**), and EdU (**f**) assays were performed to assess cell viability and proliferation; transwell assays (**g**) were used to evaluate cell migration and invasion; ImageJ software was used for cell counting. Scale bar: 50 μm. **h**–**l** qRT-PCR analysis of CDKN2B-AS1, NUF2, vimentin, MMP9, MMP2, AXIN-1, N-cadherin, and E-cadherin expression in tumor tissues of BALB/c nude mice subcutaneously injected in the flank with ACHN cells pre-infected with the lentiviral vector (*n* = 5 per group); β-actin was used as the internal control (**h**, **l**); growth curves of tumors in the nude mice (**i**); representative images (**j**), and weight of the xenografts derived from the nude mice (**k**). Error bars represent the SEM, **p < 0.01 vs. si-NC; *p < 0.05 vs. si-NC or si-NC + pcDNA3.1-vector or pGLV3-shNC + pLenti-vector; ^#^*p* < 0.05 vs. si-CDKN2B-AS1 mix + pcDNA3.1-vector or pGLV3-shCDKN2BAS1 + pLenti-vector. Data are representative from at least three independent experiments, *p* value calculated by independent sample *t* test (**b**, **c**), one-way ANOVA and Student–Newman–Keuls test (**d–**i, **k**, **l**).

To further explore the molecular mechanism of how lncRNA CDKN2B-AS1 contributes to the progression phenotype of KIRC, 718 and 2306 mRNAs co-expressed with CDKN2B-AS1 in KIRC were identified using the TANRIC database and the circlncRNAnet database, respectively, and 6055 mRNAs, related to KIRC survival were obtained from the OncoLnc database. Next, 270 mRNAs, co-expressed with CDKN2B-AS1 and affecting KIRC prognosis, were screened out (Supplementary Fig. S3). Meanwhile, using the LncMAP database, we obtained 54 genes that may be directly regulated by CDKN2B-AS1. By intersection with the above 270 mRNAs, we screened out four potential target genes of CDKN2B-AS1: NUF2, MICALL2, MICAL1, and AGBL2 (Fig. 3a). Interestingly, the qRT-PCR analysis showed that CDKN2B-AS1 knockdown for 48 h sharply reduced NUF2 mRNA expression, but did not affect MICALL2 or MICAL1 expression in 769-P and ACHN cells (Fig. 3b). In addition, CDKN2B-AS1 depletion significantly increased AGBL2 mRNA expression in ACHN cells, whereas, this effect was not observed in 769-P cells (Fig. 3b). Furthermore, in line with the change in mRNA expression, the depletion of CDKN2B-AS1 also decreased NUF2 protein levels (Fig. 3c). Therefore, we speculated that NUF2 may be one of the target genes of CDKN2B-AS1, and play a role in CDKN2B-AS1-mediated KIRC progression.

Subsequently, to evaluate the potential role of NUF2 in CDKN2B-AS1-mediated KIRC progression, KIRC cells were overexpressed NUF2 following CDKN2B-AS1 knockdown. As shown in Fig. 3d–g, the restoration of NUF2 expression significantly ameliorated the inhibitory effects of CDKN2B-AS1 depletion on cell proliferation, migration, and invasion in vitro. Consistently, results of rescue experiments showed that ectopic NUF2 expression in CDKN2B-AS1-depleted ACHN cells could significantly recover the tumorigenicity and metastatic potential in vivo. Mice in this group showed similar tumor sizes and weights, and according expression level of epithelial–mesenchymal transition molecules, including vimentin, MMP9, MMP2, AXIN-1, N-cadherin, and E-cadherin, compared to mice in the control group (Fig. 3h–l). Thus, our in vitro and in vivo data confirmed that CDKN2B-AS1 is involved in KIRC progression at least partly through the regulation of NUF2, suggesting that the CDKN2B-AS1/NUF2 axis is important for the regulation of KIRC tumor growth and metastasis.

### CDKN2B-AS1 regulates NUF2 transcription by recruiting the CBP and SMYD3 epigenetic modification complex to the promoter region

**Fig. 4:**
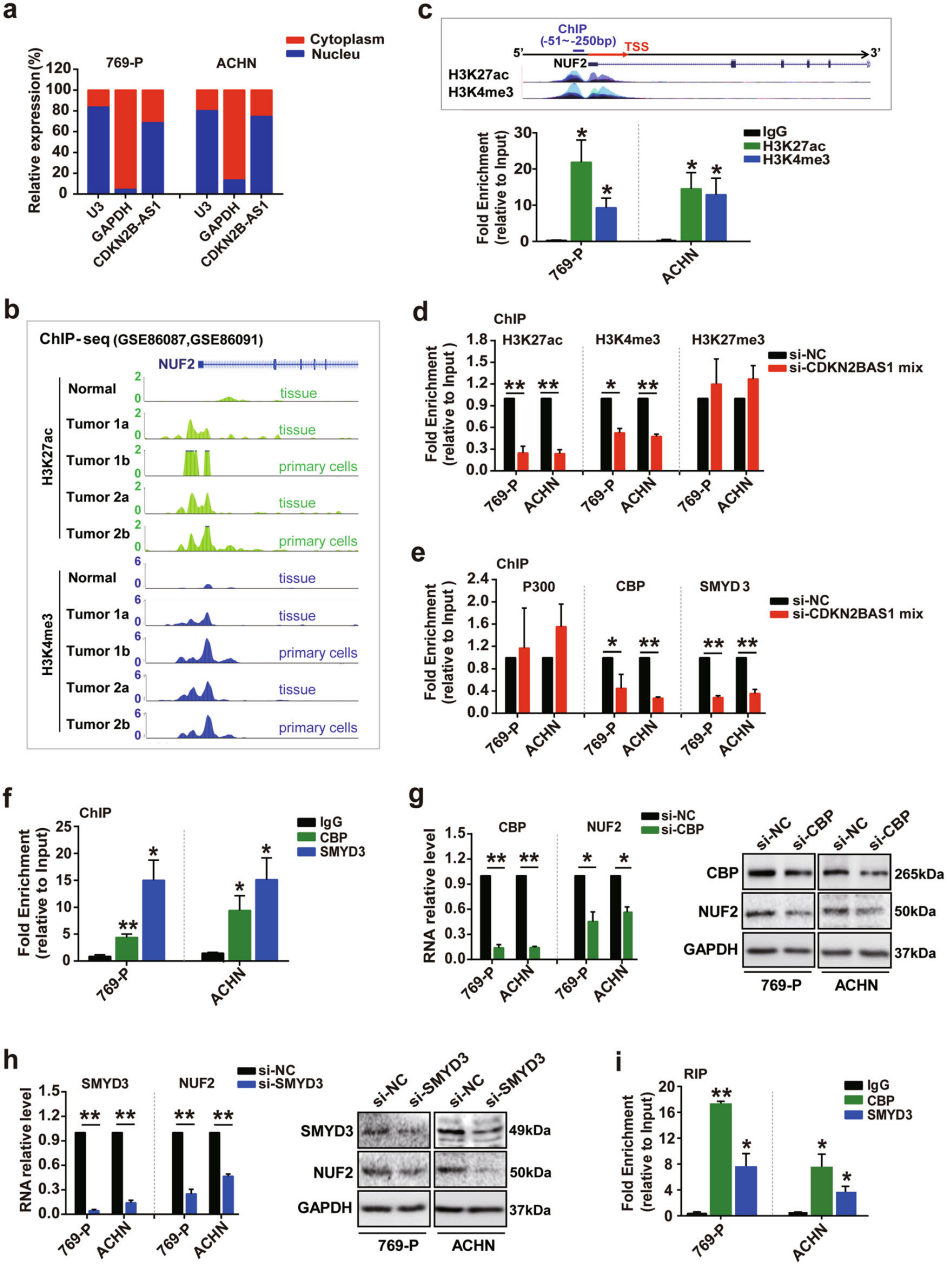
CDKN2B-AS1 regulates *NUF2* transcription by acting as a modular scaffold of CBP and SMYD3 complex to affect H3K27ac and H3K4me3 modification. **a** qRT-PCR subcellular analysis of CDKN2B-AS1 in 769-P and ACHN cells, U3 and GAPDH acted as nucleus and cytoplasm markers, respectively. **b** ChIP-seq analysis of H3K27ac and H3K4me3 enrichment at the *NUF2* promoter region in normal renal tissues, KIRC tissues, and tumor tissue-derived primary cells. **c**, **f** ChIP analysis of H3K27ac and H3K4me3 (**c**), and the chromatin-modifying enzymes CBP and SMYD3 (**f**) enrichment in the *NUF2* gene promoter; ChIP enrichment was measured using real-time PCR, normalized by the input DNA. **d**, **e** 769-P and ACHN cells were transfected with the si-CDKN2B-AS1 mix for 48 h; ChIP analysis of H3K27ac, H3K4me3, and H3K27me3 (**d**), and the chromatin-modifying enzymes P300, CBP, and SMYD3 (**e**) enrichment in the *NUF2* gene promoter; ChIP enrichment was measured using real-time PCR, normalized by the input DNA. **g**, **h** qRT-PCR and western blot analysis of CBP, SMYD3, and NUF2 mRNA and protein expression in 769-P and ACHN cells transfected with si-CBP (**g**) and si-SMYD3 (**h**) for 48 h, respectively; β-actin or GAPDH was used as the internal control, respectively. **i** RIP assay analysis of CBP and SMYD3 enrichment in CDKN2B-AS1 in 769-P and ACHN cells; RIP enrichment was measured using qRT-PCR normalized by the input RNA. Error bars represent the SEM from three independent experiments, **p < 0.01 vs. IgG or si-NC, *p < 0.05 vs. IgG or si-NC. *P* value calculated by independent sample *t* test (**d**, **e**, **g**, **h**), one-way ANOVA, and Dunnett multiple comparison test (**c**, **f**, **i**). TSS transcription start site.

Next, we examined the underlying mechanism of NUF2 regulation by CDKN2B-AS1 in KIRC. Given that the distribution of lncRNAs generally determines their function, we first performed subcellular fractionation assays to study the location of CDKN2B-AS1 in KIRC cells. CDKN2B-AS1 expression was 2.13- and 3.12-fold more abundant in the nuclear fraction compared to the cytoplasm in 769-P and ACHN cells, respectively, implying that CDKN2B-AS1 might modulate NUF2 expression during transcriptional processing (Fig. 4a). In addition, ChIP-seq data downloaded from GEO datasets showed higher H3K27ac and H3K4me3 enrichment at the *NUF2* promoter region in KIRC tissues and tumor tissue-derived primary cells compared with normal renal tissues (Fig. 4b). This was underscored by ChIP experimental findings showing H3K27ac and H3K4me3 enrichment at the *NUF2* promoter in 769-P and ACHN cells (Fig. 4c). These results could explain why NUF2 expression was increased in KIRC tissues and suggest that it may be regulated by histone epigenetic modification. Thus, we hypothesized that CDKN2B-AS1 may induce histone epigenetic alterations that regulate *NUF2* expression in KIRC.

To verify the above hypothesis, ChIP assays were performed and we observed ~75% and 42% decrease in H3K27ac and H3K4me3 levels, respectively, in the *NUF2* promoter after CDKN2B-AS1 knockdown for 48 h in 769-P cells; however, the H3K27me3 levels showed no significant changes (Fig. 4d). Correspondingly, CDKN2B-AS1 knockdown resulted in a 52% and 68% decrease in histone acetyltransferase CBP and methyltransferase SMYD3 recruitment, respectively, in the *NUF2* promoter region in 769-P cells, but did not affect acetyltransferase P300 (Fig. 4e). The effects were also confirmed in the ACHN cells (Fig. 4d, e). Furthermore, we found that CBP and SMYD3 enriched in the *NUF2* promoter, and the depletion of CBP and SMYD3 by siRNA both dramatically decreased the mRNA and protein levels of NUF2 in 769-P and ACHN cells (Fig. 4f–h). RIP assay showed an ~49- and 22-fold CDKN2B-AS1 enrichment in the anti-CBP and anti-SMYD3 immunoprecipitates in the 769-P cells, respectively, compared to the IgG control (Fig. 4i). Similar results were observed in the ACHN cells. Therefore, we concluded that CDKN2B-AS1 stimulated NUF2 transcription by interacting with the CBP and SMYD3 proteins, thereby recruiting this epigenetic modification complex to alter H3K27ac and H3K4me3 enrichment in the *NUF2* promoter region.

### CDKN2B-AS1 is stabilized by IGF2BP3 in KIRC

Although most studies on the mechanism underlying CDKN2B-AS1 regulation have mainly investigated at the transcriptional level^19^, little is known about the regulation of RNA stability, which could be affected by RNA-binding proteins. IGF2BPs, classic RNA-binding proteins, have been proven to affect the RNA stability of target genes and participate in the progression of various tumors^20^. Intriguingly, analysis of the binding potential of IGF2BPs to CDKN2B-AS1 on the RPISeq database showed that the Random Forest classifier and Support Vector Machine classifier values were both >0.5, implying possible binding between IGF2BP1, IGF2BP2, or IGF2BP3 and CDKN2B-AS1 (Supplementary Fig. S4a). Therefore, we hypothesized that the IGF2BP protein family might interact with CDKN2B-AS1 and affect its stability in KIRC.

**Fig. 5:**
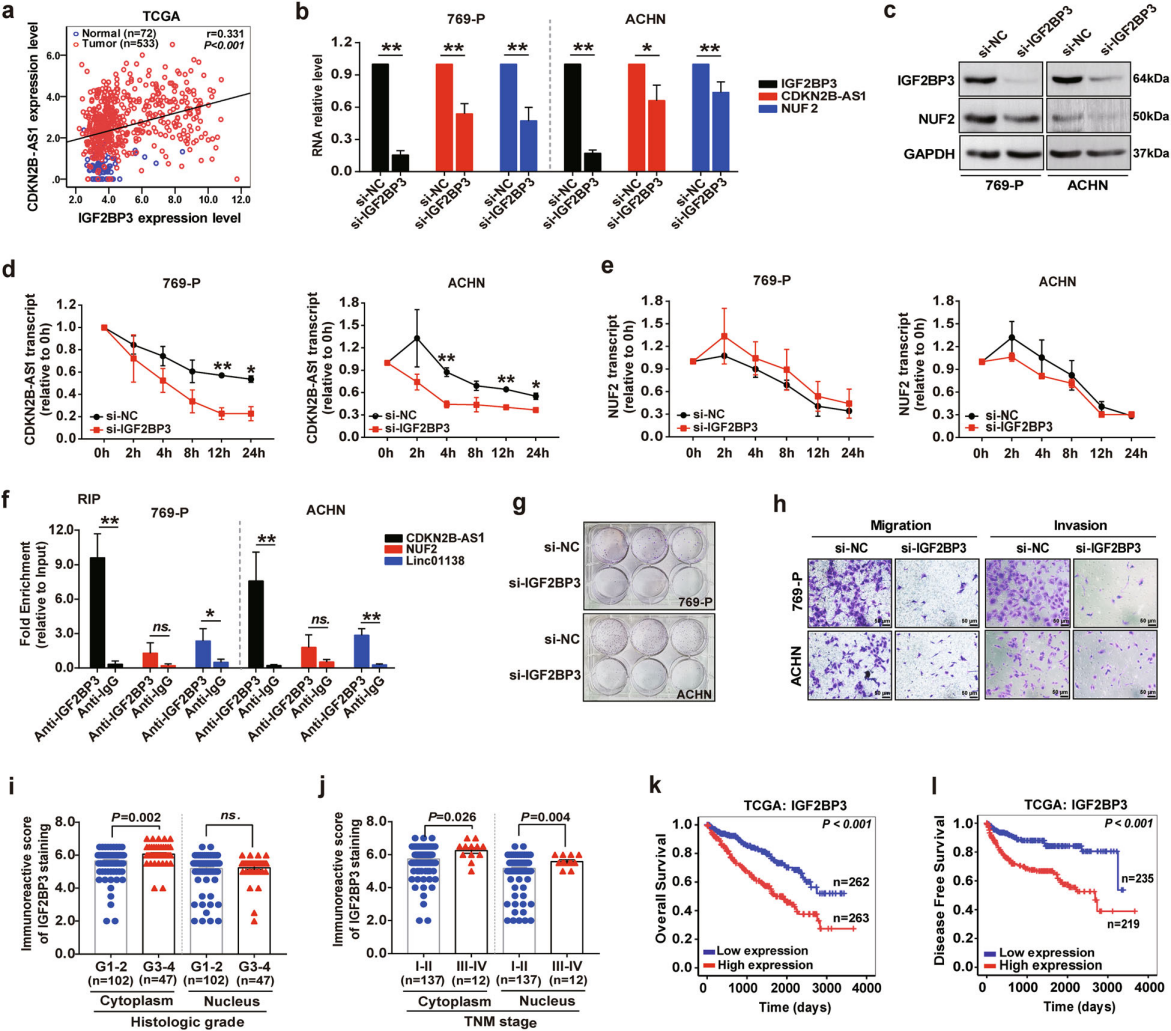
CDKN2B-AS1 is stabilized by IGF2BP3 in KIRC. **a** Pearson correlation analysis of the relationship between CDKN2B-AS1 and IGF2BP3 levels in TCGA-KIRC dataset. Normal tissues are shown as blue circles (*n* = 72) and tumor tissues as red circles (*n* = 533). **b**–**e** 769-P and ACHN cells were treated with si-IGF2BP3 for 48 h; qRT-PCR analysis of IGF2BP3, CDKN2B-AS1, and NUF2 expression with β-actin as the internal control (**b**); western blot analysis of IGF2BP3 and NUF2 protein levels with GAPDH as the internal control (**c**); qRT-PCR analysis of CDKN2B-AS1 (**d**) and NUF2 mRNA (**e**) stability, RNA levels were measured after treatment with actinomycin D (5 μg/mL) for 0, 2, 4, 8, 12, and 24 h, respectively, and compared to the level at 0 h. **f** RIP assay analysis of IGF2BP3 enrichment in CDKN2B-AS1, NUF2, and Linc01138 in 769-P and ACHN cells; RIP enrichment was measured using qRT-PCR normalized by the input RNA. **g** Colony formation assays were performed to assess cell proliferation in 769-P and ACHN cells treated with si-IGF2BP3. **h** Transwell assays were used to evaluate migration and invasion in 769-P and ACHN cells transfected with si-IGF2BP3. Scale bar: 50 μm. **i**, **j** Immunostaining score of IGF2BP3 in immunohistochemistry of patients with a different histologic grade (**i**) and TNM stage (**j**) from the paraffin-embedded KIRC tissue microarray (No. HkidE180Su02). **k**, **l** Kaplan–Meier analysis showed the overall survival and disease-free survival in KIRC patients based on the expression of IGF2BP3 in TCGA dataset; the high and low expression groups were divided by the median of IGF2BP3 levels. Error bars represent the SEM from at least three independent experiments, **p < 0.01 vs. si-NC or anti-IgG, *p < 0.05 vs. si-NC or anti-IgG. ns. no significance (*p* > 0.05). *P* value calculated by independent sample *t* test (**b**–**j**), or log-rank test (**k**, **l**).

Thus, we next sought to elucidate whether IGF2BPs could regulate the stability of CDKN2B-AS1. RNA-seq data from TCGA datasets indicated that IGF2BP2 expression in KIRC tissue was decreased and IGF2BP3 expression increased, whereas there was no significant change regarding IGF2BP1 (Supplementary Fig. S4,b). Therefore, only the change in IGF2BP3 was consistent with that of CDKN2B-AS1 in KIRC. This was confirmed by the higher immunoreactive score of IGF2BP3 staining in KIRC tissue compared with normal tissue (Supplementary Fig. S4c, d), and a significant positive correlation was noted between CDKN2B-AS1 expression and IGF2BP3 levels (Fig. 5a). Furthermore, we noticed that knockdown of IGF2BP3 observably reduced CDKN2B-AS1 and NUF2 expression in 769-P and ACHN cells (Fig. 5b, c). Interestingly, si-IGF2BP3 decreased the levels of the remaining CDKN2B-AS1 by ~58.3% and 54.5% after actinomycin D (5 μg/mL) treatment for 12 and 24 h, respectively (Fig. 5d). Similarly, we confirmed that IGF2BP3 affected CDKN2B-AS1 stability in ACHN cells (Fig. 5d). However, silencing IGF2BP3 did not affect NUF2 stability (Fig. 5e), indicating that the regulation of NUF2 expression by IGF2BP3 was carried out through the stabilization of CDKN2B-AS1. Accordingly, the RIP assay detected that the anti-IGF2BP3 antibody could dramatically enrich of CDKN2B-AS1 and another known IGF2BP3-targeting lncRNA (linc01138)^21^, but not of NUF2 (Fig. 5f). Together, our data demonstrate that IGF2BP3 interacted with and stabilized CDKN2B-AS1 in KIRC cells.

Meanwhile, the depletion of IGF2BP3 prominently inhibited KIRC cell proliferation, migration, and invasion (Fig. 5g, h). Elevated expression of IGF2BP3 was correlated with poor outcome and it could be an independent prognostic factor for worse OS in KIRC patients (Fig. 5i–l and Supplementary Tables S3–S5). Overall, IGF2BP3 may serve as an oncogene that mediates the KIRC malignant progression, similar to CDKN2B-AS1.

### IGF2BP3/CDKN2B-AS1/NUF2 axis is an attractive candidate as a KIRC prognostic and diagnostic biomarker

**Fig. 6:**
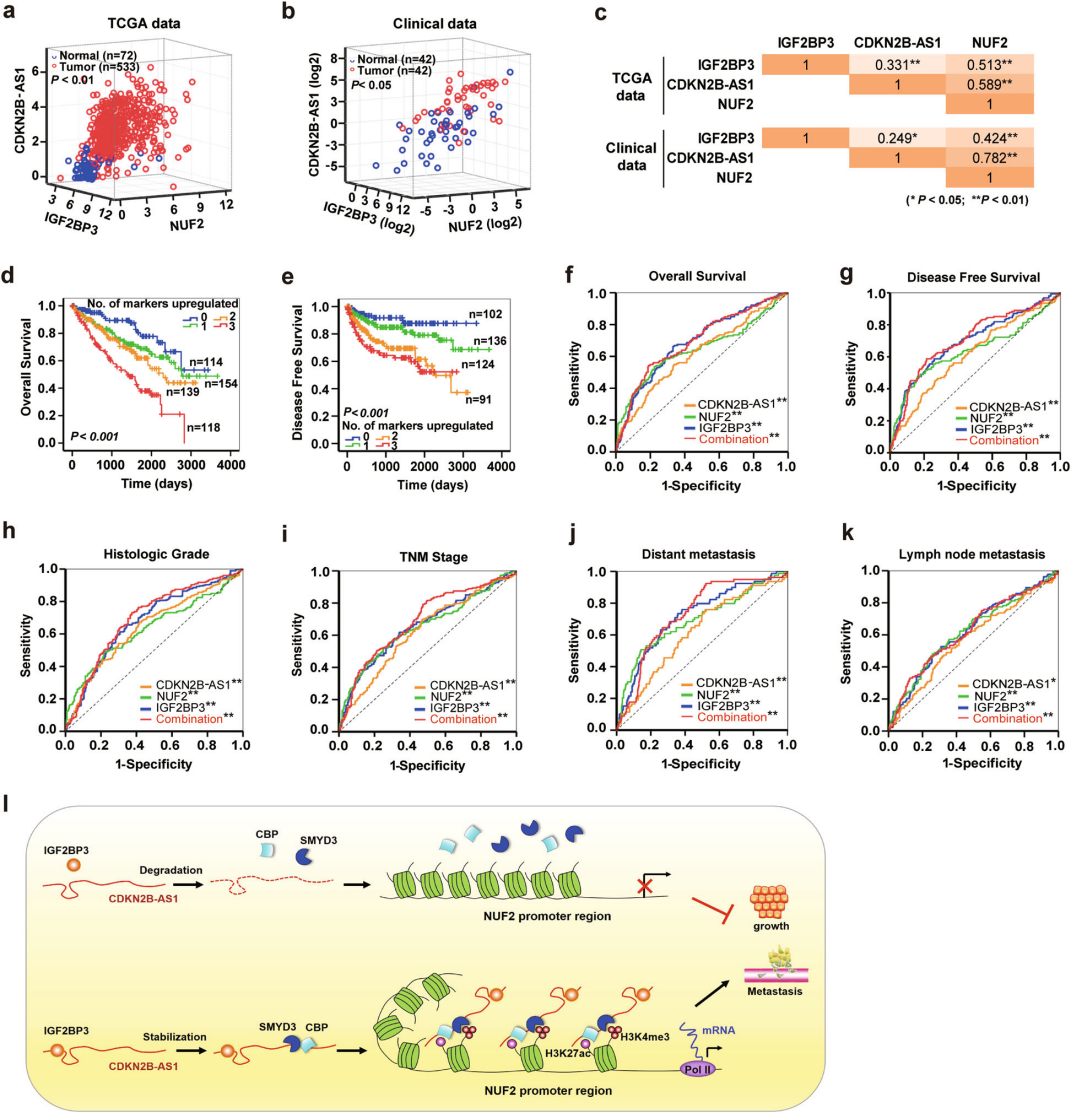
IGF2BP3/CDKN2B-AS1/NUF2 axis is an attractive candidate as a prognostic and diagnostic biomarker of KIRC. **a**–**c** Pearson correlation analysis of the relationship between CDKN2B-AS1, IGF2BP3, and NUF2 levels in TCGA-KIRC dataset (**a**) and 42 paired KIRC tissues and adjacent normal tissues (**b**), normal tissues are shown as blue circles and tumor tissues as red circles; the correlation coefficient between each two markers is shown in **c**. **d**, **e** Kaplan–Meier analysis of overall survival and disease-free survival for KIRC patients in TCGA dataset based on the number of upregulated molecular markers; CDKN2B-AS1, NUF2, and IGF2BP3 expression was stratified by the individual medians by RNA-seq data, and the patients were divided into four groups as indicated. **f**, **g** ROC curve analysis of overall survival (**f**) and disease-free survival (**g**) for CDKN2B-AS1, NUF2, and IGF2BP3 as individual biomarkers or as a combined panel (containing CDKN2B-AS1, NUF2, and IGF2BP3). **h**–**k** ROC curve analysis for CDKN2B-AS1, NUF2, and IGF2BP3 as individual biomarkers or as a combined panel to discriminate KIRC patients with high tumor grades (G3 and G4) from those with low tumor grades (G1 and G2) (**h**); KIRC patients with stages III and IV from those with stages I and II (**i**); KIRC patients with distant metastasis from patients without distant metastasis (**j**), and KIRC patients with lymph node metastasis from patients without lymph node metastasis (**k**). **p < 0.01, *p < 0.05. **l** Schematic diagram of the IGF2BP3/CDKN2B-AS1/NUF2 axis regulating tumor growth and metastasis in KIRC cells. CDKN2B-AS1 is stabilized by specifically binding to IGF2BP3 and acts as a modular scaffold of the CBP and SMYD3 epigenetic-modifying complex to increase H3K27ac and H3K4me3 modification in the promoter region of *NUF2*, thereby enhancing *NUF2* transcription, and promoting tumor cell growth and metastasis.

Based on the mechanisms described above, we proceeded to explore the prognostic and diagnostic potential of the IGF2BP3/CDKN2B-AS1/NUF2 axis in KIRC patients. Pearson correlation analysis from TCGA dataset and the clinical patient cohort showed that the levels of IGF2BP3, CDKN2B-AS1, and NUF2 were significantly positively correlated with each other in KIRC tissue (Fig. 6a–c), concordant with the above findings. Subsequently, we generated a new panel containing IGF2BP3, CDKN2B-AS1, and NUF2 to predict KIRC prognosis. Kaplan–Meier survival analysis and the log-rank test suggested that the OS and DFS gradually decreased with the increase in the number of upregulated markers, and patients with three upregulated markers showed the shortest duration of OS and DFS (Fig. 6d, e). Cox regression multivariate analysis illustrated that the number of upregulated markers could serve as an attractive predictor to evaluate the prognosis of KIRC patients (Table 1). Consistently, in the ROC analysis, the combined panel (containing IGF2BP3, CDKN2B-AS1, and NUF2) showed a higher predictive value for OS and DFS compared to any individual marker (Fig. 6f, g and Supplementary Table S6).

**Table 1:** Univariate and multivariate Cox regression analyses of upregulated marker number for overall survival in KIRC (TCGA dataset).

Variables	Univariate analysis			Multivariate analysis		
	HR	95% CI	*P* value	HR	95% CI	*P* value
Sex (male vs. female)	1.052	(0.768, 1.442)	0.752			
Age (>60 years vs. ≤60 years)	1.742	(1.271, 2.386)	0.001	1.647	(1.183, 2.292)	*0.003*
Histologic grades (G1–G4)	2.398	(1.940, 2.964)	<0.001	1.41	(1.091, 1.822)	*0.009*
TNM stages (I–IV)	1.953	(1.707, 2.236)	<0.001			
Tumor invasions (T1–T4)	1.992	(1.685, 2.355)	<0.001			
Distant metastasis (yes vs. no)	4.544	(3.303, 6.251)	<0.001			
Lymph node metastasis (yes vs. no)	1.385	(1.000, 1.918)	0.05			
Tumor size (>1.5 cm vs. ≤1.5 cm)	1.675	(1.213, 2.312)	0.002			
Number of upregulated markers (0–3)	1.585	(1.359, 1.849)	<0.001	1.22	(1.025, 1.453)	*0.025*

*HR* hazard ratio, *CI* confidence interval, *TNM* tumor node metastasis.

Moreover, ROC analysis was also performed to evaluate the diagnostic accuracy of the IGF2BP3/CDKN2B-AS1/NUF2 axis for differentiating the clinical characteristics of KIRC patients. As expected, the combined panel showed the highest accuracy in discriminating high tumor grades (G3 and G4) from low tumor grades (G1 and G2), advanced stages (stages III and IV) from early stages (stages I and II), distant metastasis from no distant metastasis, and lymph node metastasis from no lymph node metastasis (Fig. 6h–k and Supplementary Table S6). These results indicate that the IGF2BP3/CDKN2B-AS1/NUF2 axis can be used as a potential diagnostic parameter to distinguish high-risk from low-risk KIRC patients.

## Discussion

Accumulating evidence has demonstrated that CDKN2B-AS1 is upregulated in a variety of human cancers, such as bladder, gastric, and breast cancers, and serves as an oncogene that plays important roles in tumor progression^19^. Although two other studies have investigated the role of CDKN2B-AS1 in RCC and found as well that it is overexpressed^22,23^, they were limited to verify the effects of CDKN2B-AS1 in vitro experiments, and did not discuss the reasons for the upregulation of it. In the present study, we demonstrated that CDKN2B-AS1 was elevated and correlated with poor clinical outcomes in KIRC, and promoted cell proliferation, migration, and invasion in vitro. In addition, we further verified the effects of CDKN2B-AS1 on KIRC carcinogenesis in vivo and confirmed its prognostic capability by multivariate analysis. Hence, our findings, together with those of other studies^22,23^, indicate that CDKN2B-AS1 drives the malignancy of KIRC in vitro and in vivo, and has the potential to serve as an independent prognostic factor for worse KIRC progression.

CDKN2B-AS1 may drive cancer progression through several mechanisms. Canonical CDKN2B-AS1 transcriptional mechanisms may play a role, such as the in *cis* suppression of tumor suppressor genes CDKN2A and CDKN2B^24,25^, or the PRC-mediated in *trans* gene regulation^18,26^. In addition, evidence suggests that CDKN2B-AS1 acts as competing endogenous RNA (ceRNA) and as a molecular sponge for microRNA let-7a^27,28^, miR-125a^29^, miR-99a/miR-449a^30^, and miR-122-5p^31^, which are involved in the progression of prostate cancer, nasopharyngeal cancer, oral carcinoma, gastric cancer, and hepatocellular carcinoma, respectively. Moreover, CDKN2B-AS1 interacts with signaling pathways in cancers, such as p38 MAPK, PI3K/AKT, mTOR, ATM-E2F1, and TGF-β^19^. Therefore, it is not surprising that CDKN2B-AS1 can promote RCC carcinogenesis via the β-catenin pathway or function as a ceRNA for miR-141 to regulate cyclin-D1/D2 expression^22,23^. We used multiple open-source large datasets to identify the potential target genes regulated by CDKN2B-AS1, and also proved in vivo and in vitro that CDKN2B-AS1 affects KIRC tumor growth and metastasis, at least partly through NUF2, which can also serve as an oncogene to promot tumor progression^32,33^. Therefore, our study provides additional mechanistic insight into the role of CDKN2B-AS1 in KIRC progression.

The biological function of lncRNAs is largely dependent on their subcellular localization. For instance, lncRNAs in the cytoplasm can regulate the gene expression at the posttranscriptional level, including by acting as ceRNAs and protecting the target mRNAs from repression^34,35^. However, lncRNAs located in the nucleus can participate in the gene regulation at the chromatin modification and gene transcription levels^36^. Although the latest study^23^ showed that CDKN2B-AS1 can function as a ceRNA to regulate cyclin-D1/D2 expression, resulting in RCC aggressiveness, by using cell cytoplasm/nuclear fractionation assays, we found that CDKN2B-AS1 is mainly localized in the nucleus, as in previous reports on other tumors^30,37,38^, and indicating that CDKN2B-AS1 might regulate NUF2 expression via a transcriptional mechanism. Since CDKN2B-AS1 was reported to cooperate with chromatin-modifying enzymes CBX7/PRC1 or SUZ12/PRC2, it affects histone H3K27me3 enrichment in the promoter region, thus activating epigenetic activation or gene silencing^37,39^. As ChIP-seq data and ChIP-qPCR assay showed that gene-activated markers H3K27ac and H3K4me3 were more enriched in the *NUF2* promoter region of KIRC, CDKN2B-AS1 may modulate *NUF2* by changing the level of histone modification in the promoter region. Following, our experiments demonstrated that CDKN2B-AS1 could enhance the recruitment of the CBP and SMYD3 epigenetic modification complex to the *NUF2* promoter region, resulting in elevated levels of histones H3K27ac and H3K4me3, thereby stimulating NUF2 expression in KIRC cells. However, unlike in other reports^37,39^, CDKN2B-AS1 did not affect H3K27me3 enrichment in the *NUF2* promoter region, suggesting that the regulatory effects of CDKN2B-AS1 on genes have tissue and cell specificity.

Until now, most studies on the mechanism underlying CDKN2B-AS1 regulation have mainly investigated at the transcriptional level. For instance, it has been found that CpG methylation impacts the binding of CTCF, SMAD3/4, and ERα to the promoter region and regulates CDKN2B-AS1 expression^19^. In addition, CDKN2B-AS1 could also be regulated by transcription factors, including E2F1, STAT1, SOX2, SP-1, TET2, and c-MYC^19^. Despite a study^40^ in 2017, reporting that Kaposi’s sarcoma-associated herpesvirus miRNAs, and its latency-associated proteins vFLIP and vCyclin could affect the stability of CDKN2B-AS1, little was known about CDKN2B-AS1 posttranscriptional regulation, especially whether it was regulated by RNA-binding proteins. In this study, we found that the RNA-binding protein IGF2BP3 interacted with and stabilized CDKN2B-AS1, resulting in augmented posttranscriptional CDKN2B-AS1 expression. This is supported by similar reports on IGF2BP3 binding to and improving the stability of target RNAs, such as the lncRNA linc01138, and HMGA2, IGF2, HMGA2, CCND1, MMP9, and CD44 mRNAs^21,41^, and participating in the progression of various tumors^20^.

IGF2BP3, as a member of the IGF2BP family, binds to and influences the localization, transportation, and stability of target RNAs and plays an important role in cell proliferation, migration, invasion, metabolism, and embryonic development^20^. IGF2BP3 is regarded as an oncofetal protein that is abnormally highly expressed in a variety of tumors, such as pancreatic, colorectal, kidney, and oral squamous cell carcinoma^41,42^. In particular, the results of this and other studies^43–46^ showed that IGF2BP3 also stimulated the progression of KIRC and that IGF2BP3 levels in tumor tissues, and peripheral blood could be used as a predictor of KIRC metastasis and clinical prognosis. In addition to a previous report on IGF2BP3 activating the NF-κB signaling pathway^43^, we also found that IGF2BP3 can mediate the occurrence and development of KIRC by interacting with and stabilizing CDKN2B-AS1, providing a more specific molecular mechanism for the biological role of IGF2BP3 in KIRC and shedding new light on the utility of IGF2BP3 in clinical practice.

In summary, we found that the upregulated lncRNA CDKN2B-AS1 acts as an essential oncogene, promotes tumor malignant progression in vitro and in vivo, and correlates with poor prognosis in KIRC. Mechanistically, CDKN2B-AS1 was stabilized by interaction with IGF2BP3, and exerted its oncogenic activity by acting as a modular scaffold of the CBP and SMYD3 epigenetic-modifying complex to the promoter region of *NUF2*, where it enhanced NUF2 transcription by increasing local H3K27ac and H3K4me3 modifications. Clinically, the IGF2BP3/CDKN2B-AS1/NUF2 axis might be an ideal therapeutic target and a promising prognostic and diagnostic indicator for KIRC. Therefore, uncovering the precise role of this regulatory axis in KIRC progression will not only increase our knowledge of lncRNA-regulated therapeutic effects in cancer and their underlying mechanisms, but also help to develop more efficient strategies to treat KIRC.

## Supplementary information

Supplementary Tables

Supplementary Figure legends

Supplementary Figure 1

Supplementary Figure 2

Supplementary Figure 3

Supplementary Figure 4

The original data from databases
