# Supplementary material for: LncRNA CDKN2B-AS1 stabilized by IGF2BP3 drives the malignancy of renal clear cell carcinoma through epigenetically activating NUF2 transcription
Source: Cell Death Dis. 2021 Feb 19;12(2):201. doi: 10.1038/s41419-021-03489-y (PMC7895987; doi:10.1038/s41419-021-03489-y)
Supplement: Supplementary file 1 — Supplementary Tables [file 41419_2021_3489_MOESM1_ESM.docx]

**Table S1. Primer sequences used in real-time RT-PCR, ChIP, si-RNA and ASO.**

| **Assay** |  | | **Primer Sequence**  **(sense 5' - 3')** | | **Primer Sequence**  **(antisense 5'- 3')** |  |
| --- | --- | --- | --- | --- | --- | --- |
| **Real time RT-PCR** | | | | |  |  |
| β-actin | | | CTGGAACGGTGAAGGTGACA | AAGGGACTTCCTGTAACAATGCA | | |
| CDKN2B-AS1 | | | GACTTCTGTTTTCTGGCCACC | TCGGGAAAGGATTCCAGCAC | | |
| Vimentin | | | GACGCCATCAACACCGAGTT | CTTTGTCGTTGGTTAGCTGGT | | |
| MMP9 | | | TGTACCGCTATGGTTACACTCG | GGCAGGGACAGTTGCTTCT | | |
| MMP2 | | | TACAGGATCATTGGCTACACACC | GGTCACATCGCTCCAGACT | | |
| AXIN-1 | | | GGTTTCCCCTTGGACCTCG | CCGTCGAAGTCTCACCTTTAATG | | |
| N-cadherin | | | GACGGTTCGCCATCCAGAC | TCGATTGGTTTGACCACGG | | |
| E-cadherin | | | CGAGAGCTACACGTTCACGG | GGGTGTCGAGGGAAAAATAGG | | |
| NUF2 | | | GGAAGGCTTCTTACCATTCAGC | GACTTGTCCGTTTTGCTTTTGG | | |
| MICALL2 | |  | TGTGGTCCAGAGGAGGAATGA | CAGCTCCGGTGGTAAAGCC | | |
| MICAL1 | |  | GGCACTCGGTGCTAAGAAGTT | CCCCAGTGAATTTCCACCCC | | |
| AGBL2 | |  | AGTAAACCCACGTCTTCGAGA | GCCCACTCAATATGATGGATGTT | | |
| GAPDH | |  | GCACCGTCAAGGCTGAGAAC | TGGTGAAGACGCCAGTGGA | | |
| U3 snoRNA | |  | CCACGAGGAAGAGAGGTAGC | CACTCAGACCGCGTTCTCTC | | |
| CBP | |  | CACAGCCTCTCAGTCAACATC | CCTGGCGTAACTCCTCTGG | | |
| SMYD3 | |  | CGCGTCGCCAAATACTGTAGT | CAAGAAGTCGAACGGAGTCTG | | |
| IGF2BP3 | |  | CCAAGCTAGACAAGCACTAGAC | GCGGCCATTTCATCAGGGA | | |
| Linc01138 | |  | TATTTACGAAAGCTGAAAGCG | CTGCATGGGATAGGAGAAAC | | |
| **ChIP** | | |  |  | | |
| NUF2 | |  | CTTCAACTCGGACCCCGAAA | CAGGTGGGACTGGAGTTGTC | | |
| **si-RNA (ASO)** | | |  |  | | |
| CDKN2B-AS1  ASO1 | | | ACGCCTCTGACGCGACATCT |  | | |
| ASO2 | | | GCCTCATTCTGATTCAACAG |  | | |
| ASO3 | | | CTAACTCCAAAGAAACCATC |  | | |
| siRNA1 | | | GGTCATCTCATTGCTCTAT |  | | |
| siRNA2 | | | GAATGTCAGTTTTGAACTA |  | | |
| siRNA3 | | | TCCCGAGTCAGTACTGCTT |  | | |
| SMYD3 mix siRNA1 | | | AGTATCTCTTTGCTCAATCAA |  | | |
| siRNA2 | | | CAAGTATGGAAGGAAGTTCAA |  | | |
| CBP | | | CCATTTCTCCTTCCCGAATTT |  | | |
| IGF2BP3 | | | CGGTGAATGAACTTCAGAA |  | | |

**Table S2. Data for ChIP-seq downloaded from the GEO datasets.**

| **No.** | **Dataset** | **Antibody** | **Sample type** | **Tissue No.** |
| --- | --- | --- | --- | --- |
| 1 | GSM2293421 | H3K27ac | Normal tissue | 12364284 |
| 2 | GSM2293438 | H3K27ac | Tumor tissue | 86049102 |
| 3 | GSM2293314 | H3K27ac | Primary cells | 86049102 |
| 4 | GSM2293430 | H3K27ac | Tumor tissue | 40911432 |
| 5 | GSM2293319 | H3K27ac | Primary cells | 40911432 |
| 6 | GSM2293423 | H3K4me3 | Normal tissue | 12364284 |
| 7 | GSM2293428 | H3K4me3 | Tumor tissue | 40911432 |
| 8 | GSM2293315 | H3K4me3 | Primary cells | 40911432 |
| 9 | GSM2293436 | H3K4me3 | Tumor tissue | 86049102 |
| 10 | GSM2293310 | H3K4me3 | Primary cells | 86049102 |

| **Table S3. Correlation between the expression of CDKN2B-AS1/IGF2BP3 and clinicopathological features in TCGA-KIRC dataset.** | | | | | | | | |  |
| --- | --- | --- | --- | --- | --- | --- | --- | --- | --- |
| **Variable** | **Categorization** | **No. of analysis (%)** | **CDKN2B-AS1 level ^§^** | ***P* value** |  | **IGF2BP3 level ^§^** | ***P* value** | |  |
|  |  |  |  |  |  |  |  |  |  |
| **Age** |  |  |  | 0.972 |  |  | 0.572 | |  |
|  | ≤60 years | 259 (49.0%) | 2.78 ± 1.11 |  |  | 4.96 ± 2.03 |  | |  |
|  | ＞60 years | 270 (51.0%) | 2.78 ± 1.12 |  |  | 5.06 ± 2.11 |  | |  |
| **Sex** |  |  |  | 0.645 |  |  | 0.049 | |  |
|  | Male | 341 (64.6%) | 2.76 ± 1.12 |  |  | 5.15 ± 2.10 |  | |  |
|  | Female | 187 (35.4%) | 2.81 ± 1.10 |  |  | 4.78 ± 2.00 |  | |  |
| **Tumor size** | |  |  | *<0.001* |  |  | 0.214 | |  |
|  | ≤1.5cm | 255 (48.2%) | 2.57 ± 1.12 |  |  | 4.90 ± 2.10 |  | |  |
|  | ＞1.5cm | 274 (51.8%) | 2.98 ± 1.07 |  |  | 5.12 ± 2.03 |  | |  |
| **Histologic Grade** | |  |  | *<0.001* |  |  | *<0.001* | |  |
|  | G1 | 13 (2.5%) | 2.26 ± 0.81 |  |  | 3.38 ± 0.50 |  | |  |
|  | G2 | 228 (43.8%) | 2.52 ± 1.00 |  |  | 4.47 ± 1.83 |  | |  |
|  | G3 | 204 (39.2%) | 2.96 ± 1.09 |  |  | 5.18 ± 1.98 |  | |  |
|  | G4 | 75 (14.5%) | 3.21 ± 1.31 |  |  | 6.47 ± 2.35 |  | |  |
| **TNM Stage** | |  |  | *<0.001* |  |  | *<0.001* | |  |
|  | I | 264 (50.0%) | 2.53 ± 1.00 |  |  | 4.44 ± 1.67 |  | |  |
|  | II | 57 (10.8%) | 2.97 ± 1.16 |  |  | 4.94 ± 1.94 |  | |  |
|  | III | 126 (23.9%) | 2.97 ± 1.11 |  |  | 5.27 ± 2.20 |  | |  |
|  | IV | 81 (15.3%) | 3.18 ± 1.25 |  |  | 6.55 ± 2.31 |  | |  |
| **Tumor invasion** | | |  | *<0.001* |  |  | *<0.001* | |  |
|  | T1 | 269 (50.9%) | 2.54 ± 1.00 |  |  | 4.46 ± 1.70 |  | |  |
|  | T2 | 69 (13.1%) | 3.01 ± 1.11 |  |  | 5.12 ± 2.03 |  | |  |
|  | T3 | 179 (33.9%) | 3.02 ± 1.15 |  |  | 5.62 ± 2.24 |  | |  |
|  | T4 | 11 (2.1%) | 3.40 ± 1.80 |  |  | 7.99 ± 2.68 |  | |  |
| **Distant metastasis** | |  |  | *0.002* |  |  | *<0.001* | |  |
|  | No | 422 (84.2%) | 2.69 ± 1.08 |  |  | 4.78 ± 1.93 |  | |  |
|  | Yes | 79 (15.8%) | 3.11 ± 1.22 |  |  | 6.50 ± 2.31 |  | |  |
| **Lymph node metastasis** | | |  | *0.021* |  |  | *<0.001* | |  |
|  | No | 388 (74.5%) | 2.72 ± 1.08 |  |  | 4.79 ± 1.95 |  | |  |
|  | Yes | 133 (25.5%) | 2.98 ± 1.20 |  |  | 5.72 ± 2.29 |  | |  |
| ^§^Data were shown as mean ± SD; TNM: Tumor node metastasis. | | | | |  |  | |  | |

| **Table S4. Univariate and multivariate Cox regression analyses of clinical factors for overall survival in KIRC (TCGA dataset).** | | | | | | | | | | | | | | | |  |  |
| --- | --- | --- | --- | --- | --- | --- | --- | --- | --- | --- | --- | --- | --- | --- | --- | --- | --- |
| **Variables** | **Univariate analysis** | | |  | **Multivariate analysis** | | | | | | | | | | | | |
|  | **HR** | **95%CI** | ***P* value** |  | **Model 1 (CDKN2B-AS1)** | | | |  | | **Model 2 (IGF2BP3)** | | | |  |  |  |
|  |  |  |  |  | **HR** | **95%CI** | ***P* value** | |  | | | **HR** | **95%CI** | | ***P* value** | |  |
| **Sex (Male *vs.* Female)** | 1.052 | (0.768, 1.442) | 0.752 |  |  |  |  | |  | | |  |  | |  | |  |
| **Age (＞60 years *vs.*≤60 years )** | 1.742 | (1.271, 2.386) | *0.001* |  | 1.602 | (1.153, 2.226) | *0.005* | |  | | | 1.669 | (1.198, 2.324) | | *0.002* | |  |
| **Histologic Grade (G1-G4)** | 2.398 | (1.940, 2.964) | *<0.001* |  | 1.469 | (1.144, 1.887) | *0.003* | |  | | | 1.356 | (1.049, 1.752) | | *0.020* | |  |
| **TNM Stage (I-IV)** | 1.953 | (1.707, 2.236) | *<0.001* |  |  |  |  | |  | | |  |  | |  | |  |
| **Tumor invasion (T1-T4)** | 1.992 | (1.685, 2.355) | *<0.001* |  |  |  |  | |  | | |  |  | |  | |  |
| **Distant metastasis (Yes *vs.* No)** | 4.544 | (3.303, 6.251) | *<0.001* |  |  |  |  | |  | | |  |  | |  | |  |
| **Lymph node metastasis (Yes *vs.* No)** | 1.385 | (1.000, 1.918) | 0.050 |  |  |  |  | |  | | |  |  | |  | |  |
| **Tumor size ( ＞1.5cm *vs.*≤1.5cm)** | 1.675 | (1.213, 2.312) | *0.002* |  |  |  |  | |  | | |  |  | |  | |  |
| **CDKN2B-AS1 level** | 1.421 | (1.231, 1.641) | *<0.001* |  | 1.174 | (1.002, 1.375) | *0.047* | |  | | |  |  | |  | |  |
| **IGF2BP3 level** | 1.261 | (1.181, 1.346) | *<0.001* |  |  |  |  | |  | | | 1.125 | (1.041, 1.215) | | *0.003* | |  |
| **Abbreviations:** HR: hazard ratio, CI: confidence interval, TNM: Tumor node metastasis. | | | | | | | |  | |  | | | |  |  |  |  |

| **Table S5. Correlation between the protein level of IGF2BP3 and clinicopathological features**  **in KIRC (Tissue microarray: No. HKidE180Su02).** | | | | | | | | | |  |
| --- | --- | --- | --- | --- | --- | --- | --- | --- | --- | --- |
| **Variable** | **Categorization** | **No. of analysis (%)** | **IGF2BP3 protein (Immunoreative score)** | | | | | | |  |
|  |  |  | **Cytoplasm ^§^** | ***P* value** |  | **Nucleus ^§^** | | | ***P* value** |  |
| **Age** |  |  |  | 0.426 |  |  | | | 0.037 |  |
|  | ≤60 years | 94 (63.1%) | 5.81 ± 0.74 |  |  | 5.34 ± 0.67 | | |  |  |
|  | ＞60 years | 55 (36.9%) | 5.71 ± 0.82 |  |  | 4.97 ± 1.16 | | |  |  |
| **Sex** |  |  |  | 0.534 |  |  | | | 0.787 |  |
|  | Male | 106 (71.1%) | 5.75 ± 0.77 |  |  | 5.19 ± 0.95 | | |  |  |
|  | Female | 43 (28.9%) | 5.84 ± 0.78 |  |  | 5.23 ± 0.74 | | |  |  |
| **Tumor size** | |  |  | 0.105 |  |  | | | 0.729 |  |
|  | ≤4 cm | 75 (50.3%) | 5.67 ± 0.86 |  |  | 5.23 ± 0.86 | | |  |  |
|  | ＞4 cm | 74 (49.7%) | 5.88 ± 0.66 |  |  | 5.18 ± 0.93 | | |  |  |
| **Histologic Grade** | |  |  | *0.002* |  |  | | | 0.763 |  |
|  | G1-2 | 102 (68.5%) | 5.64 ± 0.80 |  |  | 5.19 ± 0.96 | | |  |  |
|  | G3-4 | 47 (31.5%) | 6.06 ± 0.61 |  |  | 5.23 ± 0.75 | | |  |  |
| **TNM Stage** | |  |  | *0.026* |  |  | | | *0.004* |  |
|  | I-II | 137 (91.9%) | 5.73 ± 0.77 |  |  | 5.17 ± 0.92 | | |  |  |
|  | III-IV | 12 (8.1%) | 6.25 ± 0.58 |  |  | 5.58 ± 0.36 | | |  |  |
| ^§^Data were shown as mean ± SD, TNM: Tumor node metastasis. | | | | | | |  |  |  |  |

| **Table S6. ROC curve analysis of the prognostic and diagnostic potential of IGF2BP3/**  **CDKN2B-AS1/NUF2 axis in KIRC (TCGA dataset).** | | | | |  |
| --- | --- | --- | --- | --- | --- |
|  | **AUC** | | **95% CI** | ***P* value** | |
| **Overall Survival** | |  | |  | |
| CDKN2B-AS1 | 0.618 | | (0.565, 0.671) | <0.001 | |
| NUF2 | 0.646 | | (0.590, 0.701) | <0.001 | |
| IGF2BP3 | 0.691 | | (0.641, 0.740) | <0.001 | |
| Combination | 0.695 | | (0.646, 0.745) | <0.001 | |
| **Disease-Free Survival** | |  | |  | |
| CDKN2B-AS1 | 0.614 | | (0.560, 0.668) | <0.001 | |
| NUF2 | 0.639 | | (0.581, 0.697) | <0.001 | |
| IGF2BP3 | 0.694 | | (0.642, 0.746) | <0.001 | |
| Combination | 0.706 | | (0.655, 0.757) | <0.001 | |
| **Histologic Grade (G3-4 *vs*. G1-2)** | |  | |  | |
| CDKN2B-AS1 | 0.643 | | (0.596, 0.690) | <0.001 | |
| NUF2 | 0.634 | | (0.586, 0.681) | <0.001 | |
| IGF2BP3 | 0.677 | | (0.630, 0.723) | <0.001 | |
| Combination | 0.699 | | (0.654, 0.745) | <0.001 | |
| **TNM Stage (III-IV *vs*. I-II)** | |  | |  | |
| CDKN2B-AS1 | 0.624 | | (0.575, 0.673) | <0.001 | |
| NUF2 | 0.653 | | (0.603, 0.702) | <0.001 | |
| IGF2BP3 | 0.658 | | (0.609, 0.707) | <0.001 | |
| Combination | 0.691 | | (0.644, 0.738) | <0.001 | |
| **Distant metastasis (Yes *vs.* No)** | |  | |  | |
| CDKN2B-AS1 | 0.620 | | (0.553, 0.687) | 0.001 | |
| NUF2 | 0.694 | | (0.624, 0.763) | <0.001 | |
| IGF2BP3 | 0.722 | | (0.660, 0.783) | <0.001 | |
| Combination | 0.734 | | (0.677, 0.790) | <0.001 | |
| **Lymph node metastasis (Yes *vs.* No)** | |  | |  | |
| CDKN2B-AS1 | 0.573 | | (0.516, 0.631) | 0.012 | |
| NUF2 | 0.621 | | (0.563, 0.678) | <0.001 | |
| IGF2BP3 | 0.622 | | (0.567, 0.678) | <0.001 | |
| Combination | 0.623 | | (0.566, 0.680) | <0.001 | |
| **Abbreviations:** AUC: area under the curve, Combination: containing IGF2BP3, CDKN2B-AS1 and NUF2, TNM: Tumor node metastasis. | | | | |  |
