# Supplementary material for: LncRNA CDKN2B-AS1 stabilized by IGF2BP3 drives the malignancy of renal clear cell carcinoma through epigenetically activating NUF2 transcription
Source: Cell Death Dis. 2021 Feb 19;12(2):201. doi: 10.1038/s41419-021-03489-y (PMC7895987; doi:10.1038/s41419-021-03489-y)
Supplement: Supplementary file 2 — Supplementary Figure legends [file 41419_2021_3489_MOESM2_ESM.docx]

**Supplementary figure** **legends**

**Figure S1. Expression of CDKN2B-AS1 in KIRC cell lines.** qRT-PCR analysis of CDKN2B-AS1 expression in KIRC cell lines: Caki-1, Caki-2, 786-O, ANCH and 769-P, β-actin was used as the internal control. Error bars represent the SEM from three independent experiments.

**Figure S2. Depletion of CDKN2B-AS1 suppresses KIRC cell proliferation.** **a** qRT-PCR analysis of CDKN2B-AS1 expression in 769-P and ACHN cells transfected with siRNAs (siRNA1, siRNA2, siRNA3) and ASOs (ASO1, ASO2, ASO3) against CDKN2B-AS1 for 48 h, respectively, β-actin was used as the internal control. **b** Cell Counting Kit‑8 assay was used to assess cell viability in 769-P and ACHN cells transfected with ASOs and siRNAs for 48 h. **c, d** Colony formation and EdU assays were performed to test cell proliferation in 769-P cells transfected with ASOs and siRNAs, respectively. Based on these pre-experimental results, we will mix ASO1, ASO3, siRNA1, and siRNA3 together (si-CDKN2BAS1 mix) for CDKN2B-AS knockdown in subsequent experiments. Error bars represent the SEM from three independent experiments. ***p* < 0.01 *vs*. ASO-NC, **p* < 0.05 *vs*. ASO-NC; ^##^*p* < 0.01 *vs*. siRNA-NC, ^#^*p* < 0.05 *vs*. siRNA-NC. Scale bar: 50 μm.

**Figure S3. mRNAs co-expressed with CDKN2B-AS1 and related to survival in KIRC.** Venn diagram showing the mRNAs that are co-expressed with CDKN2B-AS1 from the ANRIC database (718 mRNAs), or circlncRNAnet database (2306 mRNAs), the mRNAs related to survival (6055 mRNAs) from the OncoLnc database, or all (270 mRNAs) in KIRC.

**Figure S4. Expression of IGF2BP3 in KIRC tissues.** **a** RNA-Protein Interaction Prediction database was used to analyze the binding potential of IGF2BP1, IGF2BP2, and IGF2BP3 to CDKN2B-AS1. **b** IGF2BPs expression was analyzed in KIRC tissues (n = 533) and normal tissues (n = 72) using RNA-seq data in TCGA dataset, *ns.*: no significance. **c, d** Immunostaining for IGF2BP3 in tumor tissues (Tumor) and adjacent normal tissues (Normal) from the paraffin-embedded KIRC tissue microarray (No. HkidE180Su02); the immunostaining score of IGF2BP3 was calculated in (d). **e, f** Immunostaining for IGF2BP3 in patients with a different histologic grade (e) and TNM stage (f) from the paraffin-embedded KIRC tissue microarray (No. HkidE180Su02). Scale bar: 50 μm.
