# Supplementary figures and images for: LncRNA CDKN2B-AS1 stabilized by IGF2BP3 drives the malignancy of renal clear cell carcinoma through epigenetically activating NUF2 transcription
Source: Cell Death Dis. 2021 Feb 19;12(2):201. doi: 10.1038/s41419-021-03489-y (PMC7895987; doi:10.1038/s41419-021-03489-y)

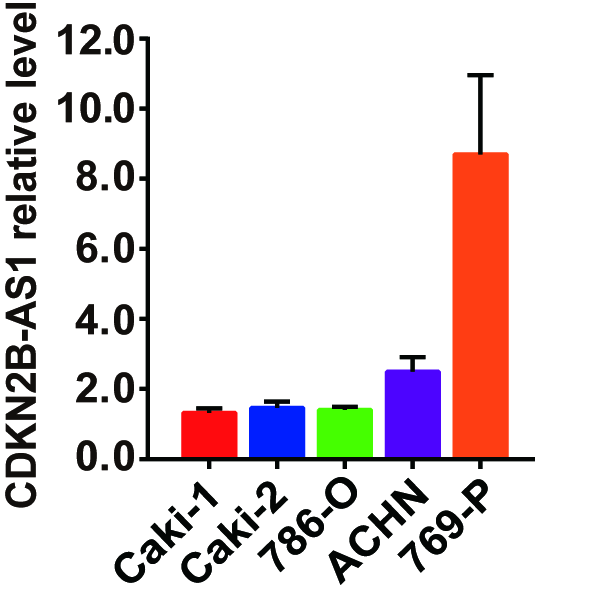

Supplement: Supplementary file 3 — Supplementary Figure 1 [file 41419_2021_3489_MOESM3_ESM.tif]

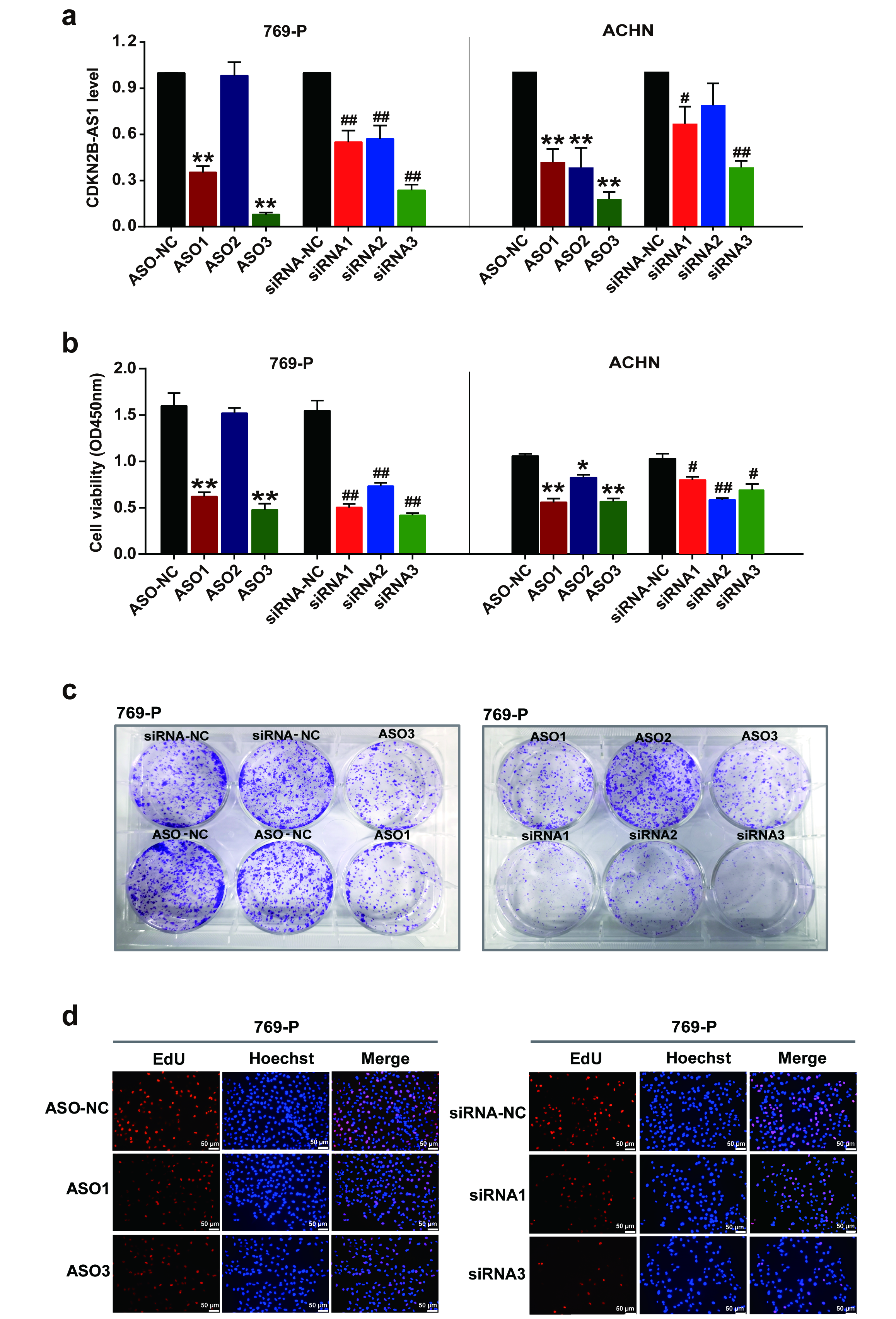

Supplement: Supplementary file 4 — Supplementary Figure 2 [file 41419_2021_3489_MOESM4_ESM.tif]

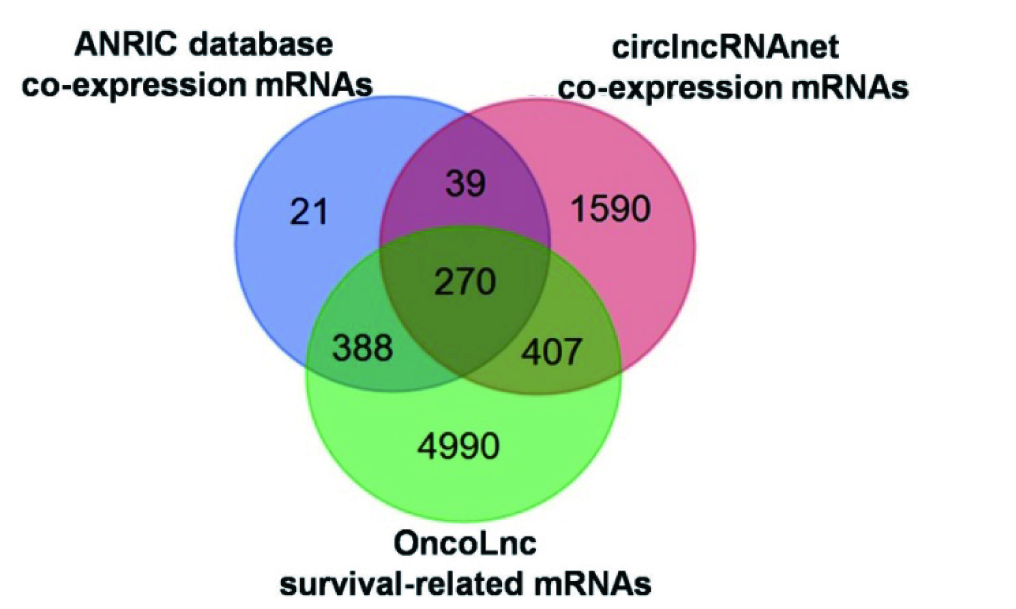

Supplement: Supplementary file 5 — Supplementary Figure 3 [file 41419_2021_3489_MOESM5_ESM.tif]

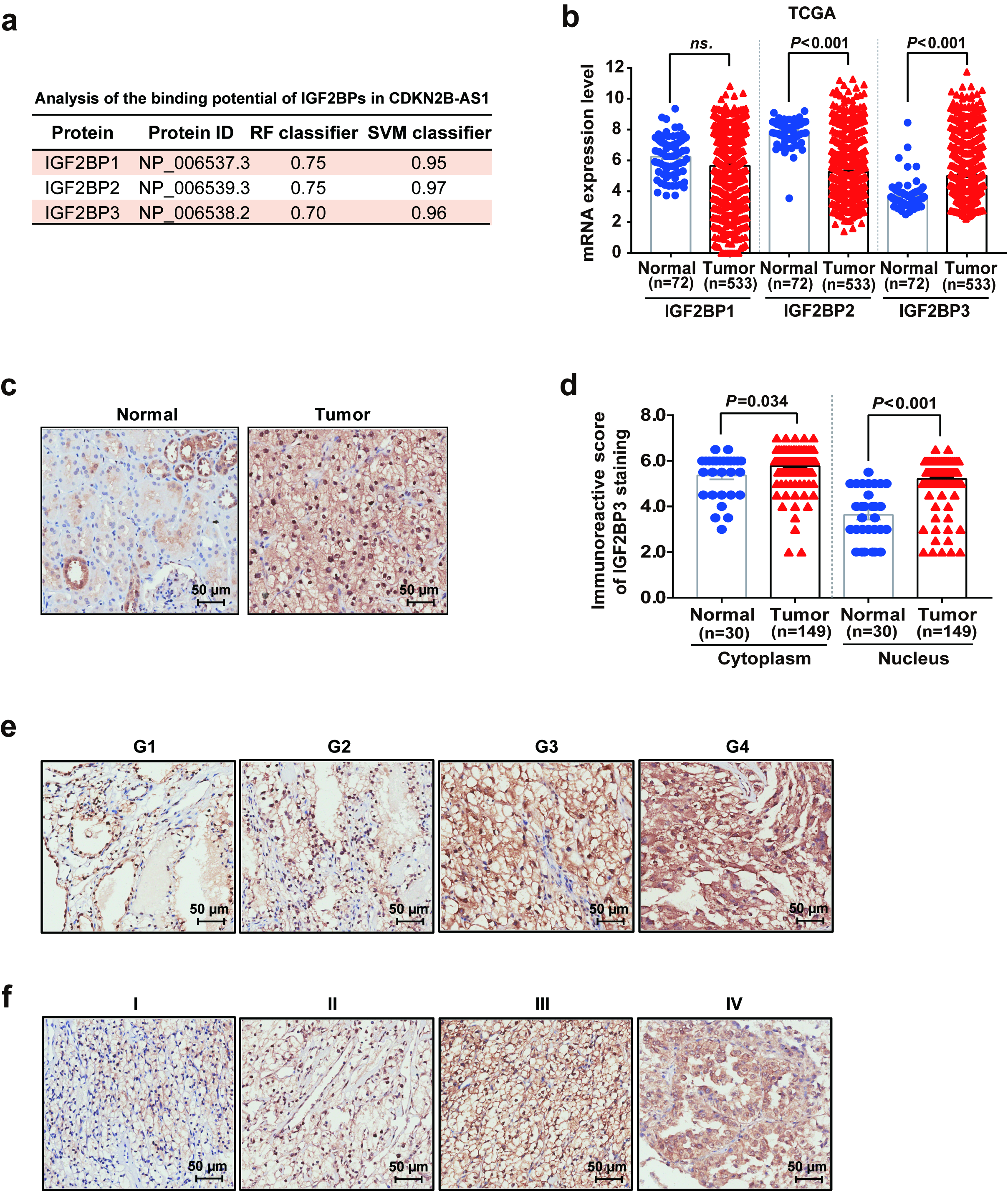

Supplement: Supplementary file 6 — Supplementary Figure 4 [file 41419_2021_3489_MOESM6_ESM.tif]
